# Receptor-like Kinases (LRR-RLKs) in Response of Plants to Biotic and Abiotic Stresses

**DOI:** 10.3390/plants11192660

**Published:** 2022-10-10

**Authors:** Aigerim Soltabayeva, Nurbanu Dauletova, Symbat Serik, Margulan Sandybek, John Okoth Omondi, Assylay Kurmanbayeva, Sudhakar Srivastava

**Affiliations:** 1 Biology Department, School of Science and Humanities, Nazarbayev University, Astana 010000, Kazakhstan; 2 International Institute of Tropical Agriculture, Lilongwe P.O. Box 30258, Malawi; 3 Department of Biotechnology and Microbiology, L.N. Gumilyov Eurasian National University, Astana 010000, Kazakhstan; 4 NCS-TCP, National Institute of Plant Genome Research, New Delhi 110067, India

**Keywords:** abiotic stress, biotic stress, stress tolerance, LRR-RLK receptors, *Arabidopsis*

## Abstract

Plants live under different biotic and abiotic stress conditions, and, to cope with the adversity and severity, plants have well-developed resistance mechanisms. The mechanism starts with perception of the stimuli followed by molecular, biochemical, and physiological adaptive measures. The family of LRR-RLKs (leucine-rich repeat receptor-like kinases) is one such group that perceives biotic and abiotic stimuli and also plays important roles in different biological processes of development. This has been mostly studied in the model plant, *Arabidopsis thaliana*, and to some extent in other plants, such as *Solanum lycopersicum*, *Nicotiana benthamiana*, *Brassica napus*, *Oryza sativa*, *Triticum aestivum*, *Hordeum vulgare*, *Brachypodium distachyon*, *Medicago truncatula*, *Gossypium barbadense*, *Phaseolus vulgaris*, *Solanum tuberosum*, and *Malus robusta*. Most LRR-RLKs tend to form different combinations of LRR-RLKs-complexes (dimer, trimer, and tetramers), and some of them were observed as important receptors in immune responses, cell death, and plant development processes. However, less is known about the function(s) of LRR-RLKs in response to abiotic and biotic stresses. Here, we give recent updates about LRR-RLK receptors, specifically focusing on their involvement in biotic and abiotic stresses in the model plant, *A. thaliana*. Furthermore, the recent studies on LRR-RLKs that are homologous in other plants is also reviewed in relation to their role in triggering stress response processes against biotic and abiotic stimuli and/or in exploring their additional function(s). Furthermore, we present the interactions and combinations among LRR-RLK receptors that have been confirmed through experiments. Moreover, based on GENEINVESTIGATOR microarray database analysis, we predict some potential LRR-RLK genes involved in certain biotic and abiotic stresses whose function and mechanism may be explored.

## 1. Introduction

Biotic and abiotic stresses have detrimental effects on growth and development in plants that are at risk of biotic and abiotic stresses. To counteract these adversities, plants have developed diverse stimuli and activation strategies. The receptors in plants are one of the primary components of plant–environment interaction that transduces the information and makes the plant aware of its surroundings. In plants, a large number of different types of receptor-like kinases (RLKs) have evolved, and they are classified based on their kinase domain and extracellular domain sequences [1,2,3]. Among the RLKs, the biggest group are the Leucine-rich-repeats–RLKs (LRR-RLKs), which have an extracellular domain LRR motif that facilitates the binding of ligands (proteins, signaling peptides, hormones, etc.). In addition to their regulatory role in plant development, shoot and root apical meristem, xylem differentiation, and BL (Brassinolide) pathways, they also play a role in the immune system and activation of cell death [4,5,6,7,8,9]. A small number of LRR-RLKs have been shown to be involved in the abiotic stress response of plants [2,10,11,12]. It is mainly achieved through the investigation of *LRR-RLK* mutant lines by inserting tDNA fragments into their exon [8,13,14], intron, UTR (untranslated region), or into the promoter region [8,15,16] and then testing them under different stresses (Tables S1 and S2). There are plenty of genetic tools that are used for the investigation of LRR-RLK gene function, but these studies were mostly conducted in model plant *Arabidopsis thaliana*, and thereafter in tomato (*Solanum lycopersicum*) and tobacco (*Nicotiana benthamiana*). The ubiquitous presence of LRR-RLK with varying sequences across the plant genera underscores the need for its investigation in other plants in order to expand the knowledge and thereby to understand their role more specifically. RLKs were classified into different groups based on their functions, such as growth and development processes and biotic and abiotic stress responses (reviewed Yuriko Osakabe, and Beg Hab Kim review articles [2,17]). Some of the well-studied *LRR-RLK*s, such as *BRASSINOSTEROID INSENSITIVE 1 (BRI1)* or *SOMATIC EMBRYOGENESIS RECEPTOR KINASE 3 (SERK3)*; *SOMATIC EMBRYOGENESIS RECEPTOR KINASE 1, 2*, and *4 (SERK1/2/4); BRI1-ASSOCIATED RECEPTOR KINASE 1 (BAK1); BAK1-INTERACTING RECEPTOR-LIKE KINASE*1 (*BIR1*); and *SUPPRESSOR OF BIR1-1* (*SOBIR1), ELONGATION FACTOR-Tu (EF-Tu) RECEPTOR (EFR)* were discussed as being essential for immune responses [2,17,18]. Credible evidence has been provided to demonstrate that some of the RLKs, such as *RECEPTOR-LIKE PROTEIN KINASE1* (*RPK1)* and *RECEPTOR-LIKE KINASE 7 (RLK7* or other name *LRR XI-23)*, are involved in water stress [2,16], and *PHLOEM INTERCALATED WITH XYLEM-LIKE 1 (PXL1)* in cold stress [19]. Notably, some of the above-mentioned *LRR-RLKs*, such as *BAK1/SERK3; SERK1,-2*, and -*4*; and *BRI1*, share the same signaling pathways in Mitogen-Activated Protein Kinase (MAPK) activation, Ca^2+^ influx, and the production of reactive oxygen species (ROS) in order to initiate plant responses to biotic or abiotic stresses and/or developmental cues (see review [20]). Among the SERK-mediated signaling pathways, crosstalk occurs at multiple levels, and it is possible to have crosstalk with other *LRR-RLKs*. Recent *LRR-RLK* studies showed additional crosstalk of LRR-RLKs, and also new achievements for additional functions of known *LRR-RLKs*. Despite the exploration of so many *LRR-RLKs* from *A. thaliana* over a period of time, the functional role of several of these has not yet been explored.

The in silico studies have shown that LRR-RLK harbors a transmembrane domain (TM), intracellular kinase domain (KD), and LRR-containing extracellular domain (ECD) [21]. This extracellular domain of LRR–RLKs was classified according the structure of LRR, where the LRR II, III, VI, IX, X, XI, XII, and XIII sub-families contained the cysteine residues in the amino-terminal of the LRR motifs [21]. This extracellular domain could have an impact on oligomerization [21]. Moreover, it was shown that LRR-RLKs with short extracellular domains are mainly co-receptors [22], which help to hold the ligand and stabilize and enhance transduction of the intracellular signal together with the ligand-binding receptor [23,24,25]. Some of the LRR-RLKs interact with each other, which allows for the formation of different heterodimers or trimers, thereby allowing them to become multifunctional [26,27]. In vitro and in vivo studies confirmed the heterodimer complexes of LRR-RLK and few trimeric complexes [15,16,28,29,30,31,32]. These formations are important for triggering immune responses, metabolic pathways, stem development, etc. Therefore, the use of double, triple, or quadruple mutants proved to be a useful tool for achieving their function and/or interactions [16,29,30,31,32,33]. The interaction of LRR-RLKs and its mechanism was mainly studied in *A. thaliana*.

Here, we update the functioning of LRR-RLKs in response to biotic and abiotic stresses in *A. thaliana*, and in other plants as well, such as *N. benthamiana*, *S. lycopersicum*, *Oryza sativa, Triticum aestivum, Hordeum vulgare, Brachypodium distachyon, Brassica napus, Medicago truncatula, Gossypium barbadense, Phaseolus vulgaris, Solanum tuberosum*, and *Malus robusta*. Additionally, we compiled the information on genetic tools used in the investigation of LRR-RLK’s role in biotic and abiotic stress response. The Microarray data analysis (GENEINVESTIGATOR, https://genevestigator.com/, accessed on 3 August 2022) of the LRR-RLK genes, which responded to biotic and abiotic stimuli, suggested the possibility of additional functions and their cross-link in triggering different signaling processes. Furthermore, we have presented the information on stress-related LRR-RLKs as dimer, trimer, and tetramer complexes, which were experimentally achieved in *A. thaliana*. Furthermore, an ATTED databases (https://atted.jp/hclust/, accessed on 2 February 2022) analysis for protein interactions pointed towards additional possible interactions among the LRR-RLKs, however, these need to be validated experimentally [33].

## 2. Abundance of *LRR-RLKs* Genes in the Plant Genome

The abundant availability of genome and RNA sequences allowed us to identify the potential *LRR-RLK* in different plants, such as annual and perennial plants, crops, trees, herbs, etc. (Table 1). In primitive plants such as *Sedum alfredii, Selaginella moellendorffii, Amborella trichopoda*, and *Physcomitrella patens*, there are about 60–134 genes. In higher plants, the identified *LRR-RLK* gene numbers vary from 200 to 600 (*LRR-RLK* genes) (See Table 1). It was shown that algae do not contain any *LRR-RLK* genes similar to plants and it was suggested that the structural combination of LRRs and kinase domains (KD) to form new genes may have occurred after the divergence of land plants from green algae [34]. An in silico analysis revealed that the expansion in *LRR-RLKs* is a result of tandem and segmental duplication events (Table 1). Additionally, the exon/intron compositions and motif arrangements were considerably conserved among members in the same groups or subgroups in plants (Table 1).

The research on *LRR-RLKs* were mainly conducted in *A. thaliana* due to the availability of a large dataset. The increase in DNA and RNA sequencing in different crops and *A. thaliana* allowed for the identification of the numbers of stress-related *LRR-RLKs* (Table S1) and their sequence in other plants such as *O. sativa, G. max, M. truncatula, Populus*, *V. vinifera*, *S. lycopersicum*, *B. napus*, and *Z. mays* (Table S2). Note that some of the stress-related LRR-RLKs in *A. thaliana* have bigger numbers of orthologs in some plants, such as *G. max, M. truncatula, O. sativa*, and *Z. mays*, as compared to *A. thaliana* (Table S2). Only a few orthologs of stress-related *LRR-RLK* genes were investigated under biotic and abiotic stresses and their functions were elucidated in different plants by using different mutants of *LRR-RLKs* (Table S3).

## 3. Stress-Related *LRR-RLKs* in Plants

**Figure 1:**
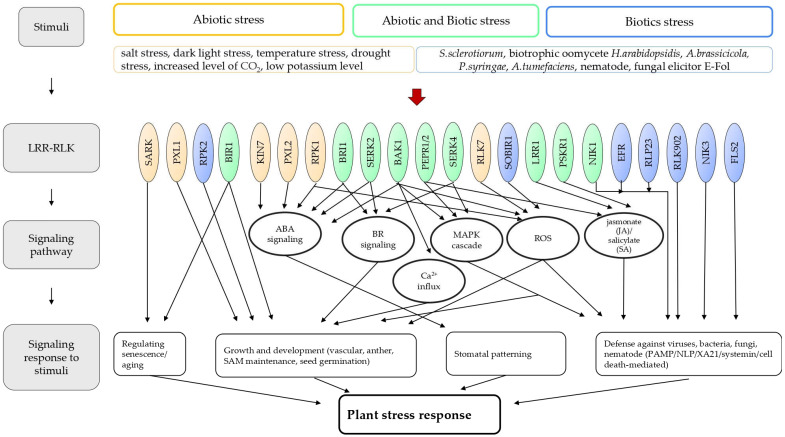
**Scheme of signaling pathways triggered by biotic- and abiotic-tested LRR-RLKs and crosstalk among them.** The LRR-RLKs regulate various plant processes, including growth, development, and responses to biotic and abiotic stresses. After perceiving abiotic and/or biotic stimuli by the LRR-RLKs, activation of diverse signaling, such as MAPK activation (BAK1, PEPR1/2, SERK4), Ca^2+^ influx (BAK1), reactive oxygen species (ROS) production (RPK1, BAK1, RLK7, SOBIR1), BR signaling (BRI1, SERK2, SERK4), ABA signaling (KIN7, PXL2, RPK1, BRI1, SERK2, BAK1), jasmonate (JA), and salicylate (SA) (PEPR1/2, LRR1, PSKR1) occurs. Some LRR-RLKs may activate several signaling pathways depending on the type of stimuli: RPK1-ABA and ROS pathways; BRI and SERK2—ABA and BR pathway; PEPR1/2—MAPK and JA/SA pathway, SERK4—BR and MAPK pathway, BAK1-BR signaling, Ca^2+^ and MAPK pathways. Senescence may be associated with SARK and BIR1. Defense mechanisms against biotic stress stimuli are mediated by JA, SA, ROS, and MAPK pathways, and also additionally followed by LRR-RLKs: NIK1, EFR, RLP23, RLK902, NIK3, and FSL2. Yellow color indicates tested abiotic stimuli, blue color is for tested biotic stimuli, and green for both stimuli.

From a pool of 223 *LRR-RLKs* identified in *A. thaliana (*Table 1*)*, some were clustered as regulators of various growth and development processes (stem cell maintenance, anther development, determination of the fate of a cell and organ development, cell expansion, stem stomata development) [67,68,69,70,71], while others were shown or suggested to be important in biotic and abiotic stress responses (Figure 1). One of the extensively studied genes among *LRR-RLKs* is *BAK1/SERK3*, which regulates multiple processes such as Brassinosteroid (BR) signaling, growth and development, and stomatal patterning, and also activates the expression of antimicrobial proteins [20,72]. Recently, the role of BAK1 in guard cell ABA signaling was demonstrated and *bak1* mutants showed more loss of water as compared to wild-type. The ABA increased the formation of a complex between BAK1 and OPEN STOMATA1 (OST1) near the plasma membrane [73]. In addition, *bak1–5* mutants revealed that the post-invasive resistance of *A. thaliana* to *Alternaria brassicicola* is independent of pathogen-triggered indole-3-carboxylic acid and its derivatives (ICAs) and camalexin biosynthesis [74]. Notably, the function of BAK1/SERK3 in the immune response was shown in tobacco and tomato plants against the late blight pathogen (*Phytophthora infestans*) and bacterial and nematode infection [75,76]. Other *SERKs*, such as *SERK1* and *SERK2*, were also important genes for conferring resistance against bacterial leaf blight and fungal infection, respectively [20,77,78]. In rice, *OsSERK2* was shown as a positive regulator of immunity, interacting with the rice immune receptor kinases (XA21 and XA3) [78]. It was suggested that *SERK2* could be a target for sRNAs of *Sclerotinia sclerotiorum* and the resulting action may contribute to the silencing of immune components in plants [79]. Recently, using the *serk2* mutant lines, it was observed that *SERK2* is a component of BR signaling and it regulates BR signaling and salt resistance in rice [80]. Previously, *SERK4* together with *SERK3* were shown to trigger a series of defense responses [20], but in recent studies using knock-out and overexpressing lines of *SERK4*, it was observed that *SERK4* negatively regulates the leaves senescence process [81].

Another well-studied LRR-RLK gene is *BRI1*, which interacts with *SERK3* by mediating BR signaling through the BRI1/BAK1 complex [72], and it regulates stem elongation, vascular differentiation, seed size, fertility, flowering time, and senescence [8,17,82]. Moreover, the mutant of *BRI1* shows pleiotropic effects on disease resistance along with plant development regulation [83], and it displays ABA-hypersensitive primary root growth [84,85]. In *B. napus*, *S. lycopersicum*, and *B. distachyon*, the role of *BRI1* in BR signaling was confirmed [86,87,88]. Furthermore, *BRI1* was involved in BR signaling through MAPK and Ca^2+^-dependent protein kinases in rice [89]. Additionally, it was suggested that *SlBRI1* is related with systemin-mediated systemic defense response [90], however it was not established conclusively [87]. In tobacco, *NbBRI1* was involved in BR-mediated systemic defense signaling by regulating H_2_O_2_ and NO production [91]. Recently, *BRI1* manipulation in different cereals resulted in drought tolerance [86], and disease resistance [83]. Additionally, the overexpression of wheat *TaBRI1* in *A. thaliana* revealed early flowering and enhancement of seed production [92], while overexpression of SlBRI1 promoted fruit ripening and ethylene production, and increased the levels of carotenoids, ascorbic acid, soluble solids, and soluble sugars during fruit ripening [93]. In potato (*S. tuberosum*), BRI1 was involved in the regulation of tuberization, thus suggesting other possible roles of BRI1 [94]. BIR1 is another LRR-RLK that forms a complex with SERK3/BAK1 or with other SERKs, and these complexes were shown to repress the effector-triggered immunity (ETI) in the absence of pathogen effectors [72]. The LRR-RLK SOBIR1 also interacts with SERKs, serving as a stabilizer of the protein complex and aids receptor complexes in triggering defense responses [18]. Notably, the homolog of *SOBIR1* activated the immune response in tomato against fungal infection [18], but not in antiviral infection [95]. In tobacco plants, *NbSOBIR1* was involved in the immunity of *N. benthamiana* through monitoring the production of ROS [96]. Furthermore, the manipulation of *GbSOBIR1* in cotton (*G. barbadense)* plants resulted in resistance to *Verticillium* [97]. While EFR regulated the immune response of plants after perceiving bacterial flagellin and EF-Tu by forming complexes with SERKs [72], it was observed that *A. thaliana efr* mutants lacking EF-Tu perception are more susceptible to transformation by *Agrobacterium tumefaciens* [98]. This revealed the importance of the EF-Tu perception system for plant defense. The homologs of *EFR* in tobacco and in tomato were also involved in pathogen-associated molecular patterns (PAMP)-triggered immunity, and suggesting transgenic expression of EFR could be used as an engineering tool against broad-spectrum bacterial infections [99]. In rice, it was shown that receptors EFR and XA21 recruit similar immune signaling [100]. The overexpression of *AtEFR* in different crops shows resistance to bacterial infection and/or symptoms. In wheat and apple, the overexpression of *AtEFR* enhanced resistance against bacterial disease, fire, and blight [101], respectively. Similarly, in *M. truncatula*, it reduced the bacterium infection [102] and in potato it enhanced bacterial wilt resistance [103].

SERKs also form complexes with PHYTOSULFOKINE (PSK) RECEPTOR 1 (PSKR1), which is known to regulate root growth and hypocotyl elongation [20]. Moreover, *PSKR1* was also identified as an important component of plant defense [104]. *PSKR1* suppresses salicylate-dependent defense responses, where the *pskr1* mutants exhibit early senescence, a salicylate (SA)-associated response, and are impaired in wound healing, a jasmonate (JA)-associated response [104]. Moreover, *OsPSKR1* was involved in the immune response against bacterial leaf streak in rice through salicylic acid (SA) pathway signaling [105]. *PEP1 RECEPTOR 1 (PEPR1)* and *PEPR2* were involved in immune responses through interaction with SERK3 through AtPep1-triggered ROS production and ethylene signaling [20]. Furthermore, PEPR1 recognizes AtPep3 and increases tolerance to salt stress as well as immune response [106]. Furthermore, it was suggested that AtPeps-PEPR signaling pathway is involved in stomatal closure through an OST1-independent mechanism under biotic stress [107]. *PEPR1/2 ORTHOLOG RECEPTOR-LIKE KINASE1* from *S. lycopersicum* was shown to regulate responses to systemin, necrotrophic fungi, and insect herbivory [108]. Previously, RECEPTOR-LIKE PROTEIN KINASE1 (RPK1) was linked to the water stress response [2], wherein it regulates ABA/stress signaling by controlling ROS homeostasis. Additionally, RPK1 and BAK1 form complexes with OST1 to regulate ABA-induced stomatal closure [73,109].

The LEUCINE-RICH REPEAT PROTEIN 1 (LRR1) forms a complex with pathogenesis-related protein10 (PR10), and it leads to cell-death-mediated defense signaling [110]. Additionally, it was shown in vivo and in vitro that during plant responses to drought stress, LRR1 and KINASE 7 (KIN7) are degraded by PLANT U-BOX PROTEIN 11 (PUB11), an E3 ubiquitin ligase [111]. This KIN7 phosphorylates and activates tonoplast-located channels during ABA- and CO_2_-mediated stomatal closure [112]. Recently, Leucine-rich receptor-like kinase homologs in cereals such as barley and wheat showed important components of defense responses against *Fusarium* by disbalancing salicylic acid signaling [113]. The silencing of *LRR1* in rice in the *XA21* genetic background (*XA21-LRR1Ri*, XA21 is 21 amino acid tyrosine-sulfated epitope derived from the bacterial protein) compromises resistance to bacterial leaf blight, indicating involvement in XA21-mediated immune response [114]. Other *LRR-RLK*, RECEPTOR-LIKE KINASE 902 (RLK902) previously showed importance in resistance to *Hyaloperonospora arabidopsidis* (downy mildew) in A. thaliana [115]. RLK902 associates with ENHANCED DISEASE RESISTANCE 4 (EDR4) and with BRASSINOSTEROID-SIGNALING KINASE1(BSK1), a key component of plant immunity [116]. Not much is known about LRR-RLK-like NSP-interacting kinases (NIKs), such NIK1, NIK2, and NIK3, and their interaction with geminivirus nuclear shuttle protein (NSP). The binding of NSP to NIK inhibits its kinase activity in vitro, and a phenotypic analysis of NIKs mutant lines suggests that NIKs are involved in the antiviral defense response [117]. It was shown that *At-FEI2* (cell wall receptor-like kinase) plays a positive role in *Arabidopsis* and in tomato defense against *Botrytis cinerea* based on the study using knockout mutants of the Bc-siR37 [118].

The *LRR-RLK* genes have been shown to play important roles in response to abiotic stresses. In previous studies, *ERECTA-LIKE1 (ERL1)*, a member of the gene family closely related to LRR-RLKs, was shown to synergistically regulate plant development and morphogenesis and functions in response to abiotic stresses, especially for heat response and drought [2,119]. Recently, it was observed that the ERECTA family was involved in sensing salt and osmotic stresses [120]. Furthemore, other *LRR-RLK* genes like *PXY-Like 2 (PXL2)* have been proven to be essential in vascular development through recognizing small signaling peptides, and they play a role in ABA signaling [121]. While the *PHLOEM INTERCALATED WITH XYLEM-LIKE 1 (PXL1)* is essential in cold and heat stress (through the ROS), the PXL1 regulates HISTIDINE-RICH DEHYDRIN1 (AtHIRD1) and LIGHT-HARVESTING PROTEIN COMPLEX I (AtLHCA1) by phosphorylation [122]. Another *LRR-RLK* gene, such as *SCHENGEN 3 (SGN3*), is related with developmental processes, particularly with microdomain organization and enhanced suberization in the endodermis. Additionally, it was shown that the *sgn3* mutant was extremely sensitive to environmental conditions, such as different temperatures and nutrient deficiency [123]. Moreover, the role of BRI1-LIKE3 (BRL3) was reported in sensing glucose and flg22, where BRL3 together with REGULATOR OF G-PROTEIN SIGNALING 1 (AtRGS1) prevents excess ROS burst and control growth inhibition [124]. Additionally, the overexpression of *BRL3* resulted in drought stress tolerance in *A. thaliana* through the accumulation of osmoprotectant metabolites, such as proline and sugars [125]. In rice, the *OsBRL1* and *OsBRL3* were shown to be partly involved in BR perception in the roots [126]. Another LRR-RLK, *RLK7/LRR XI-23*, was involved in the regulation of seed germination and oxidative stress [127]. Furthermore, the LRR-RLKs, RECEPTOR-LIKE PROTEIN KINASE 2 (RPK2) also known as TOAD2) together with CLAVATA3 (CLV3), regulate the development of anthers, embryo, and stem cell homeostasis in the shoot apical meristem [2]. Single mutants of *RPK2* and *CLAVATA1* (*CLV1*) and *CLAVATA2 (CLV2)* showed importance in nematode parasitism [128]. Furthermore, RPK2 interacts with other LRR-RLK, such as SENESCENCE-ASSOCIATED RECEPTOR-LIKE KINASE (SARK)—also known as CLAVATA3 INSENSITIVE RECEPTOR KINASE 3 (CIK3)—to maintain stem cell homeostasis [129] and anther development [33]. *SARK* was shown to be a positive regulator of senescence through hormone imbalances [130]. Interestingly, *PpSARK* in moss was shown as a positive regulator of senescence in salt stress responses and was also suggested to be a negative regulator of senescence [131]. In common bean’s SARK, it did not coordinate senescence in nodules [132].

Thus, the mentioned *LRR-RLK* gene(s), which were tested in different biotic or abiotic stress/senescence processes, shared similar signaling pathways, such as reactive oxygen species (ROS) production, Ca^2+^ influx, activation of MAPK, regulation of defense genes, regulation of stomatal patterning, hormonal regulation, and regulation of senescence-related gene(s), to trigger the response of plants (See the scheme in Figure 1). Remarkably, some *LRR-RLK* genes show crosstalk in triggering different programs (Figure 1) and their numbers increased compared to the most recent review articles, which mentioned *LRR-RLK* genes in biotic and abiotic stresses [17,20]. Therefore, we have additionally collected all of the known single mutants of *LRR-RLK* genes and enlisted their phenotypic changes under experimentally tested biotic and/or abiotic stresses in Table S1, where their crosstalk can be easily observed. The information was gathered from the available microarray and RNA sequencing data (online available GENEINVESTIGATOR), which is also useful in predicting the potential role of genes for a particular stress stimulus [133]. Several *LRR-RLK* genes were identified in biotic or abiotic stresses (Table S1). The microarray data analysis of these *LRR-RLK* genes showed the alterations in expression under both types of stress stimuli i.e., biotic and abiotic (Figure S1). For example, *FEI2, NIK3, RLK902, RPK2, EFR, SOBIR1*, and *NIK1*, which were tested only in biotic stresses, showed a change in the expression under abiotic stress as well (Figure S2). Similarly, the reverse was observed for genes involved in abiotic stresses, such as *RPK1, BRL3, ERL1, PXL1, PXL2, SARK, RLK7/LRR XI-23, SGN3, KIN7*, and *PEPR1*, as it showed a change in expression for biotic stress too (Figure S3). The detailed characterization of these *LRR-RLK* genes under both stresses is likely to provide additional information on their mechanistic role and may give insight into the crosstalk among the LRR-RLKs.

## 4. Interactions among the Stress-Related LRR-LRKs

Several mutant lines, such as single, double, triple, and/or quadruple mutants, were generated (Table S1) to investigate the function of these genes [8,15,33,134,135]. In these studies, it was shown that the formation of complexes (dimer, trimer, or tetramer) is important in relaying the signals to the downstream components [13,14,28,29,30,31]. The genetic evidence for interactions was confirmed by pull-down, gel filtration, bimolecular fluorescence complementation (BiFC), co-immunoprecipitation (CoIP), and protein kinase assay, as presented in Table 2, Figures S4–S7. Most of the known functional interactions among the LRR-RLK were shown as heterodimers and, in a few cases, as homodimers, e.g., SERK 1 and SERK2 (Table 2). The trimers were also shown and they perform different functions, e.g., SOBIR-BAK1-RLP23, BON1-BAK1-BIR1, ER-BAK1-TMM, BIK1-BAK1-ERL1/2, FLS2-BAK-BIK1, and FLS2-BIK1-RBOHD play a role in the immune system [14,28,29,31,136,137]; and CLV1-CLV2-CRN in stem cell regulation [32,138]. The formation of some of the LRR-RLK complexes were dependent on ligand stimulation, for example, ligands such as flg22 [fragment of bacterial flagellin that binds the FLAGELLIN SENSITIVE 2 (FLS2) receptor] and elf18 (the N-terminal of EF-Tu) stimulate the formation of BAK-FLS2 or BAK-EFR dimers, respectively [16]. Similarly, SCFE1 (SCLEROTINIA CULTURE FILTRATE ELICITOR1) or nlp20 (peptide motif) ligands stimulate the formation of the BAK1-SOBIR1-RLP23 complex [14], while the binding of AtPep1 (endogenous peptide elicitor) induces PEPR1-BAK1 heterodimerization [139]. The INFLORESCENCE DEFICIENT IN ABSCISSION (IDA) stimulate the formation of heterodimers of SERK1 and SERK2 with HAESA (HAE) and HAESA-LIKE2 (HSL2), and Cf-4 of the SERK1-SOBIR1 complex [14,30] (see Figures S4–S7). Steroids may induce the generation of a LRR-RLKs complex, such as SERKs and TETRATRICOPEPTIDE-REPEAT THIOREDOXIN-LIKE 3 (TTL3), which activate the BR signaling pathway [16,140,141], and an application of brassinolide (BL) stimulates the formation of BAK1-BRI1 [16]. However, it was shown that the formation of heterotrimers, BAK1-ER-TMM, BAK1-ERL1-TMM, and BAK1-ERL2-TMM, are not dependent on stimulation ligands (EPF1 and EPF2) [29].

The formation of heterodimers with other LRR-RLKs, so-called co-receptors that usually have short extracellular domains, is important for holding the ligand and stabilizing it for the enhancement of the transduction of the intracellular signal [22]. The role of heterodimers was mainly as a defense response, development process, BR signaling, or cell death (Figures S4–S7). For example, BRI1 generated complexes with SERKs (BAK1/SERK3, SERK1, and SERK4) and TTL3—only for BR pathway activation [16,140,141], BIR1 can interact with BAK1, SERK1, SERK2, and SERK4 to inhibit plant cell death [14,30], and EFR with SERK1, BAK1, and SERK4/BKK1 can activate the immune response [16,98] and EFR with GLYCINE-RICH PROTEIN7 (GRP7) activates the PAMP-triggered immune (PTI) response against *Pseudomonas syringae* [142]. SOBIR forms a complex with BAK1 for immune responses against *P.infestans* and *S. sclerotiorum* [14,30], and the heterotrimer with AtRLP23 is formed in the absence of BIR1 to activate cell death [14]. Additionally, PSKR1 may interact with SERK1/2 and BAK1 [143]. Furthemore, CLV1 forms heterodimers with BAM1 (BARELY ANY MERISTEM1), SARK, and CRN (CORYNE), RPK2 [32,138,144,145], which are important for apical and young floral meristem development. Conversely, other LRR-RLKs that form variant complexes were shown to perform different roles. For example, BAK1 interacts with FLS2, SOBIR1, NIK1, BKK1, BIR2, PEPR1, PSKR1, EFR, BIK1, RLP23, ERL2, ERL1, ER, and TMM to activate immune responses [14,16,28,29,30,31]; with BIR1 to inhibit plant cell death; with RPK1, BIR1, BON1 (BONZAI1), SOBIR1, and HSL2 to activate cell death [30,109,146]; with BRI to activate BR pathway [8,16]; and with ER or ERL1 to regulate stomatal patterning [147] (Figure S4). Other multifunctional LRR-RLK genes are SERK1 and SERK2, which interact with EXCESS MICROSPOROCYTES 1 (EMS1) for anther development [135]; with PXY to regulate procambial cell proliferation [23,148]; with ER or ERL1 to regulate stomatal patterning; and with FLS2 and EFR, BRI1 and BAK1 for other functions (Figure S5).

Some multifunctional LRR-RLKs, such as BAK1, SERK 1, SERK2, SERK4, SARK, NIK1, and NIK3, were classified as co-receptors based on the length of the extracellular domain (ECD) [22], which are able interact with other LRR-RLKs, and they are grouped as the LRR (II) family. Some of the examples of members belonging to a particular family are as follows: LRR (II)—SERK 4, NIK1, NIK3, SARK, and SERK5; LRR(III)—RPK1, RLK902, PRK1, PRK2A, PRK4, PRK5, PRK6, MRLK; LRR(VII)—HYDROGEN PEROXIDASE-RESISTANT 1 (GHR1); LRR(IX)—BIR1; and LRR(V)—SRF1, SRF2, SRF3, SRF5, SRF6, SRF7, and SRF8 (STRUBBELIG-RECEPTOR FAMILY 1–3, 5–8). Other LRR-RLKs such as SOBIR1, FEI1, EFR, FLS2, BRI1, CLV1, ERL1, ERL2, ER, HSL2, PXY, BAM1, BAM2, PEPR1, GSO1 (GASSHO1), PSKR1, EMS1, RPK2, BRL1, BRL2, BRL3, PSY1R (PHYTOSULFOKINE RECEPTOR), and IOS1 (IMPAIRED OOMYCETE SUSCEPTIBILITY1) (Table 2 and Table S4), which are classified as ligand-perceiving receptors with long ECD and are grouped as LRR (I), LRR (X), LRR (XI), and (LRR XII) [22]. Mainly, these co-receptors potentially bind with ligand-perceiving receptor groups for the activation and stabilization of complexes that sense signals [8,13,16,22,28]. They also enhance signaling through sequential reciprocal receptor transphosphorylation [149]. Different combinations of interactions among co-receptors with different ligand-binding LRR-RLKs allows for the substitution of each other. This creates difficulties in using a single mutant line for LRR-RLK gene studies. For example, SERK1, SERK2, and BAK1 share the same players, like EFR and FLS2, although BAK1 is preferred by FLS2 among other SERKs, whereas EFR does not show preference to BAK1 [16]. Yet, SERK1 and SERK2 could easily substitute each other for activation of a similar plant response [16]. This regulation of stability of complexes is less studied, with only a few reported cases. For example, BAK1 interacts with BIR1 or BIR2 to prevent heterodimerization of the BAK1-FLS2 complex, and thereby inhibits an immune system response [14,30]. Furthermore, BRI1 and SERK3 do not ubiquitously interact, they only show interaction in the endosomes and in restricted areas on the plasma membrane. In these sites, BAK1 is shown as a redistributor of BRI1 receptors [15]. Thus, most LRR-RLKs form complexes, and some of the LRR-RLK in the complexes play key roles in the complex, such as BAK1 [16,28], and some components in the complexes are able to substitute for each other.

Additionally, the ATTED database showed different interactions among the proteins based on various experiments and data (Table 2). The analysis of these interactions confirmed the known interactions of BAK1, SERK 1, SERK 2, SERK 4, NIK1, NIK3, and SARK (Figures S4–S7), but also the additional potential heterodimerizations for these co-receptors (Table 2). The less investigated co-receptors, like NIK1 and SARK, according to this database, have a potential for binding with BAK1. Importantly, the CIKs, such as NIK1, NIK3, and SARK, were shown in the stress response and in natural senescence [4,22], but their co-receptors’ (heterodimers or homodimer) involvement in senescence or the stress response was not shown (Table 2). It will be exciting in the future to reveal the additional functions of these CIKs and to show the new interactions of the collected LRR-RLKs mentioned in Table S4. The interactions among the LRR-RLKs in other plants are less studied, and only a few were confirmed in tomato and tobacco [75,150], rice [77], *M. truncatula* [151], and wheat [92] (Table S4), and that was mainly done by using the yeast two-hybrid assays, co-immunoprecipitation, and BiFC methods. Altogether, the study of new interactions of LRR-RLKs in *A. thaliana* and other plants and the generation of new double or triple mutants could help in functional and interactional analyses of LRR-RLKs.

**Table 2:** Experimentally proven and potential interactions of stress-related LRR-RLKs with LRR-RLK co-receptors from LRR II. Data taken from different well-studied research manuscripts and from ATTED database. The formation of heterodimers between the LRR-RLKs were labeled as “heterodimer” inside of box cross between two LRR-RLKs one from column and second from row. Below the heterodimer formation were shown the method/s of identifications heterodimer formation such as CoIP—coimmunoprecipitation; BiFC—bimolecular fluorescence complementation, gel filtration, pull-down, in vivo, genetically (by mutants analysis), kinase assay, co-sedimentation in solution, solid-phase assay. NA indicates not available data reported the formation of heterodimer/s. Grey colored box indicates that information taken from ATTED database about interaction was confirmed experimentally (published data), and white zone was not confirmed experimentally.

LRR-RLK	SERK2	SERK1	SERK4/BKK1	SERK3/BAK1	SARK/Cik3	NIK1	NIK3/Cik1	References
**co-receptors** (LRR-RLKs from **LRR II** family **accordingly classification of** [22])								
**BIR1**	heterodimer (BiFC)	heterodimer (BiFC)	heterodimer (BiFC)	heterodimer (genetically, in vivo, pull-down)	heterodimer (solid-phase assay)	heterodimer (solid-phase assay)	*NA*	[14,152]
**FLS2**	*NA*	heterodimer (in vivo)	heterodimer (in vivo)	heterodimer (in vivo, CoIP, gel filtration)	*NA*	heterodimer (in vitro, pull down)	*NA*	[16,153,154]
**ERL1**	heterodimer (CoIP)	heterodimer (CoIP)	heterodimer (CoIP)	heterodimer (in vivo, CoIP)	heterodimer (solid-phase assay)	heterodimer (solid-phase assay)	*NA*	[147,152,153]
**BRI1**	*NA*	heterodimer (genetically, in vivo)	heterodimer (in vivo)	heterodimer (genetically, in vivo, in vitro)	*NA*	*NA*	*NA*	[146,149,155]
**EFR**	*NA*	heterodimer (in vivo)	heterodimer	heterodimer (genetically, CoIP, in vivo)	heterodimer (solid-phase assay)	heterodimer (solid-phase assay)	*NA*	[16,152,156]
**ER**	heterodimer (genetically, CoIP)	heterodimer (genetically, CoIP)	heterodimer (solid-phase assay)	heterodimer (genetically, pull-down, in vivo)	heterodimer (solid phase assay)	heterodimer (solid-phase assay)	*NA*	[29,147,152,153]
**PXY**	heterodimer (genetically, in vivo, in vitro)	heterodimer (genetically, in vivo, in vitro)	*NA*	heterodimer (genetically, in vivo, in vitro)	heterodimer (solid-phase assay)	heterodimer (solid-phase assay)	heterodimer (solid-phase assay)	[23,153]
**co-receptors** (LRR-RLKs from **LRR II** family **accordingly classification of** [22])								
**HSL2**	heterodimer (CoIP)	heterodimer (CoIP)	*NA*	heterodimer (CoIP)	heterodimer (solid-phase assay)	heterodimer (solid-phase assay)	*NA*	[152,157]
**BAK1**	heterodimer (genetically, CoIP)	heterodimer (BiFC, CoIP)	heterodimer (genetically)	*NA*	heterodimer (solid-phase assay)	heterodimer (genetically, in vivo, in vitro)	heterodimer (solid-phase assay)	[127,130,131,132]
**SERK1**	heterodimer (genetically, in vivo)	homodimer (genetically, in vivo)	heterodimer (solid-phase assay)	heterodimer (BiFC, CoIP)	heterodimer (solid-phase assay)	heterodimer (solid-phase assay)	*NA*	[152,153,155,158]
**SERK2**	homodimer (genetically, in vivo)	heterodimer (genetically, in vivo)	*NA*	*NA*	*NA*	*NA*	heterodimer (solid-phase assay)	[152,158]
**EMS1**	heterodimer (genetically, kinase assay, BiFC)	heterodimer (genetically, kinase assay, BiFC)	*NA*	*NA*	*NA*	*NA*	*NA*	[135]
**SERK4**	heterodimer (genetically, CoIP)	heterodimer (solid-phase assay)	*NA*	heterodimer (genetically)	Heterodimer (solid-phase assay)	Heterodimer (solid-phase assay)	*NA*	[16,152]
**NIK1**	*NA*	heterodimer (solid-phase assay)	heterodimer (solid-phase assay)	Heterodimer (genetically, in vivo, in vitro)	Heterodimer (solid-phase assay)	*NA*	*NA*	[152,153,154]
**PSKR1**	heterodimer (solid-phase assay, co-sedimentation in solution)	heterodimer(molecular sieving)	*NA*	heterodimer (genetically, CoIP, gel filtration)	heterodimer (solid-phase assay)	*NA*	*NA*	[143,153]
**co-receptors** (LRR-RLKs from **LRR II** family **accordingly classification of** [22])								
**SOBIR1**	heterodimer (solid-phase assay)	*NA*	*NA*	heterodimer (genetically, CoIP)	*NA*	*NA*	*NA*	[30,153]
**RPK1**	*NA*	*NA*	*NA*	heterodimer (genetically, pull-down, kinase assay)	heterodimer (solid-phase assay)	heterodimer (solid-phase assay)	*NA*	[109,152]
**PEPR 1**	*NA*	*NA*	*NA*	heterodimer (pull-down, gel filtration)	*NA*	*NA*	heterodimer (solid-phase assay)	[153,159]
**ERL2**	*NA*	heterodimer (solid-phase assay)	heterodimer (solid-phase assay)	heterodimer (genetically)	heterodimer (solid-phase assay)	heterodimer (solid-phase assay)	*NA*	[29,152]
**RPK2**	*NA*	*NA*	*NA*	*NA*	heterodimer (in vivo, pull-down)	heterodimer (solid-phase assay)	heterodimer (genetically)	[129,153]
**CLV1**	*NA*	*NA*	*NA*	*NA*	heterodimer (in vivo, pull-down)	*NA*	heterodimer (genetically)	[129]
**BAM1**	heterodimer (solid-phase assay)	*NA*	*NA*	*NA*	heterodimer (pull-down)	heterodimer (solid-phase assay)	heterodimer (genetically, BiFC, CoIP)	[33,153]
**BAM2**	*NA*	*NA*	*NA*	*NA*	heterodimer (pull-down)	heterodimer (solid-phase assay)	heterodimer (genetically, BiFC, CoIP)	[33,153]

## 5. Conclusions

Plants must evolve to adapt and tolerate harsh environments. The perception of biotic and abiotic stimuli is crucial for the survival of plants. Among the large numbers of receptors, the LRR-RLK are not only involved in different development biological processes but also in stress response processes. The rapid development of genome and RNA sequence analyses have allowed for the identification of the many LRR-RLKs genes in different monocot and dicot plants. As compared to *A. thaliana*, not much is known about the functions of LRR-RLKs in other plants such as tomato, tobacco, wheat, rice, *H.vulgare, B. distachyon*, etc. Additionally, stress-related LRR-RLKs in *A. thaliana* are mainly shown to play a role in biotic stresses, and to some extent in abiotic stresses. For the functional study of LRR-RLKs, a single mutant is of little use to capture all their functions, and, as LRR-RLKs can be substituted by other members, they do not have non-specific ligand binding and they have the ability to make different protein complexes (di-, tri-, or tetramer). The double, triple, and quadruple mutant generation for LRR-RLKs in *A. thaliana* and/or crop plants is a powerful tool for identifying the function role of LRR-RLKs.

## Figures and Tables

**Table 1:** Number of *LRR-RLK*s present in different plants. Whole genome sequence data of the enlisted plant species was analyzed for the identification of LRR-RLKs. The identified LRR-RLK genes (numbers) with their expansion characteristic (presence of tandem duplication, motif conservation, and duplication) were based on different studies. Genome size for each species was given. Mb indicates mega base pairs. “-” indicates no data available.

Numbers of *LRR-RLK*	Plant Species	Genome Size	Presence of Tandem Duplication	LRR-RLK Organizations	References
**0**	*Chlamydomonas reinhardtii*	120 Mb	-	-	[34]
**60**	*Sedum alfredii*	39.1 Mb	-	motif conservation	[35]
**67–81**	*Selaginella moellendorffii*	100 Mb	present	motif conservation	[34]
**94**	*Amborella trichopoda*	870 Mb	present	motif conservation	[36]
**134**	*Physcomitrella patens*	500 Mb		-	[34,37]
**176**	*Phoenix dactylifera*	658 Mb	present	-	[37,38]
**180**	*Cucumis sativus*	367 Mb	present	segmental duplication	[37,39]
**201**	*Fragaria vesca*	240 Mb	present	-	[40]
**211**	*Medicago truncatula*	465 Mb	present	exon/intron organization, motif conservation	[37,41]
**215**	*Vitis vinifera*	500 Mb	present	segmental duplication	[37,42]
**226**	*Arabidopsis thaliana*	133 Mb	present	-	[37,43]
**227**	*Jatropha curcas*	320 Mb	present	-	[37,44]
**230–236**	*Vernicia fordii* *Vernicia montana*	1310 Mb	Present (*V. fordii*)	motif conservation (both), segmental duplications (*V. fordii*)	[45,46]
**239**	*Solanum lycopersicum*	900 Mb	present	-	[37,47]
**247**	*Theobroma cacao*	430 Mb	present	motif conservation	[37,48]
**250**	*Zea mays*	2400 Mb	present	random chromosomal distribution	[37,49]
**267**	*Prunus mume*	280 Mb	present	-	[40]
**268**	*Solanum tuberosum*	840 Mb	present	lineage-specific expansion	[37,50]
**268**	*Prunus persica*	265 Mb	present	-	[37,40]
**292**	*Raphanus sativus*	574 Mb	present	motif conservation	[51]
**298**	*Gossypium arboreum*	1750 Mb	present	conserved exon/intron organization	[52]
**297–300**	*Citrus clementina* *Citrus sinensis*	370 Mb 380 Mb	present	-	[53]
**300**	*Brassica rapa*	455 Mb	present	intron/exon pattern organization, motif conservation	[37,54]
**310**	*Setaria italica*	515 Mb	present	motif conservation, segmental duplication	[37,55]
**312**	*Brassica nigra*	522 Mb	present	intra-chromosomal duplication, conserved loci duplication	[56]
**332**	*Oryza sativa*	430 Mb	present	exon duplication, mutation, and exon shuffling	[37,57]
**367**	*Glycine latifolia*	939 Mb	present	-	[58]
**384**	*Gossypium raimondii*	761 Mb	present	motif conservation	[37,59]
**427**	*Pyrus bretschneideri*	512 Mb	present	exon/intron organization, motif conservation	[60]
**441**	*Populus trichocarpa*	500 Mb	present	segmental duplication, exon/intron organization and motif conservation	[37,61]
**484**	*Brassica juncea*	920 Mb	present	segmental duplications, intra-/inter-genomic duplications	[62]
**485**	*Malus domestica*	750 Mb	present	-	[37,40]
**494**	*Glycine max*	1100–1150 Mb	present	exon/intron organizations, motif arrangements	[37,63]
**531**	*Triticum aestivum*	17,000 Mb	present	segmental duplications, intra-genomic duplications	[64]
**548**	*Arachis hypogaea*	2700 Mb	present	segmental duplication, exon/intron organization and motif conservation	[65]
**589**	*Thinopyrum elongatum*	4780 Mb	present	segmental duplications	[66]

## Data Availability

Data is contained within the article or supplementary material.

## Supplementary Materials

The following are available online at https://www.mdpi.com/article/10.3390/plants11192660/s1, Figure S1: Microarray analysis of the biotic and abiotic stress related LRR-RLK genes transcripts in response to biotic and abiotic stresses in WT (Col ecotype). Figure S2. Microarray analysis of the biotic stress related LRR-RLK genes transcripts in response to biotic and abiotic stresses in WT (Col ecotype). Figure S3. Microarray analysis of the abiotic stress related LRR-RLK genes transcripts in response to biotic and abiotic stresses in WT (Col ecotype). Figure S4. Protein interactions of BAK1/SERK3 with other LRR-RLK with experimental proved functional role. Figure S5. Protein interactions of SERK1 and SERK4 with other LRR-RLK with experimental proved functional role. Figure S6. Protein interactions of SERK2 with other LRR-RLK with experimental proved functional role. Figure S7. Protein interactions of CIK’s (NIK1, NIK3 and SARK) with other LRR-RLK with experimental proved functional role. Table S1. Phenotypes of single mutants of LRR-RLK genes in *A. thaliana* tested under different biotic and abiotic stresses. Table S2. Orthologous of stress related LRR-RLKs genes in *A. thaliana, O. sativa, G. max, M. truncatula, Populus*, *V. vinifera*, *S. lycopersicum*, *B. napus*, *Z. mays*. Table S3. Genetic tools in different crop plants in investigation of stress-related LRR-RLKs. Table S4. Potential interactions of stress-related LRR-RLKs (from LRR II family) with other LRR-RLKs. References [160,161,162,163,164,165,166,167,168,169,170,171,172,173,174,175,176,177,178,179,180,181] are cited in the supplementary materials.

## References

[1] Gish L.A., Clark S.E. (2011). The RLK/Pelle Family of Kinases. Plant J..

[2] Osakabe Y., Yamaguchi-Shinozaki K., Shinozaki K., Tran L.S.P. (2013). Sensing the Environment: Key Roles of Membrane-Localized Kinases in Plant Perception and Response to Abiotic Stress. J. Exp. Bot..

[3] Dievart A., Gottin C., Peacuterin C., Ranwez V., Chantret N. (2020). Origin and Diversity of Plant Receptor-like Kinases. Annu. Rev. Plant Biol..

[4] Dievart A., Clark S.E. (2004). LRR-Containing Receptors Regulating Plant Development and Defense. Development.

[5] Wang J., Kucukoglu M., Zhang L., Chen P., Decker D., Nilsson O., Jones B., Sandberg G., Zheng B. (2013). The Arabidopsis LRR-RLK, PXC1, Is a Regulator of Secondary Wall Formation Correlated with the TDIF-PXY/TDR-WOX4 Signaling Pathway. BMC Plant Biol..

[6] Wang R., Ji Y., Wang J., Yang S., Song Y. (2015). Vascular Expression of Populus LRR-RLK Genes and the Effects of Their Overexpression on Wood Formation. Mol. Breed..

[7] Li X., Salman A., Guo C., Yu J., Cao S., Gao X., Li W., Li H., Guo Y. (2018). Identification and Characterization of LRR-RLK Family Genes in Potato Reveal Their Involvement in Peptide Signaling of Cell Fate Decisions and Biotic/Abiotic Stress Responses. Cells.

[8] Li J., Wen J., Lease K.A., Doke J.T., Tax F.E., Walker J.C. (2002). BAK1, an Arabidopsis LRR Receptor-like Protein Kinase, Interacts with BRI1 and Modulates Brassinosteroid Signaling. Cell.

[9] Zhou J., Wang P., Claus L.A.N., Savatin D.V., Xu G., Wu S., Meng X., Russinova E., He P., Shan L. (2019). Proteolytic Processing of Serk3/Bak1 Regulates Plant Immunity, Development, and Cell Death. Plant Physiol..

[10] Park S.J., Moon J.C., Park Y.C., Kim J.H., Kim D.S., Jang C.S. (2014). Molecular Dissection of the Response of a Rice Leucine-Rich Repeat Receptor-like Kinase (LRR-RLK) Gene to Abiotic Stresses. J. Plant Physiol..

[11] Liu X.S., Liang C.C., Hou S.G., Wang X., Chen D.H., Shen J.L., Zhang W., Wang M. (2020). The LRR-RLK Protein HSL3 Regulates Stomatal Closure and the Drought Stress Response by Modulating Hydrogen Peroxide Homeostasis. Front. Plant Sci..

[12] Lin F., Li S., Wang K., Tian H., Gao J., Zhao Q., Du C. (2020). A Leucine-Rich Repeat Receptor-like Kinase, OsSTLK, Modulates Salt Tolerance in Rice. Plant Sci..

[13] Albert I., Böhm H., Albert M., Feiler C.E., Imkampe J., Wallmeroth N., Brancato C., Raaymakers T.M., Oome S., Zhang H. (2015). An RLP23-SOBIR1-BAK1 Complex Mediates NLP-Triggered Immunity. Nat. Plants.

[14] Gao M., Wang X., Wang D., Xu F., Ding X., Zhang Z., Bi D., Cheng Y.T., Chen S., Li X. (2009). Regulation of Cell Death and Innate Immunity by Two Receptor-like Kinases in Arabidopsis. Cell Host Microbe.

[15] Russinova E., Borst J.W., Kwaaitaal M., Caño-Delgado A., Yin Y., Chory J., De Vries S.C. (2004). Heterodimerization and Endocytosis of Arabidopsis Brassinosteroid Receptors BRI1 and AtSERK3 (BAK1). Plant Cell.

[16] Roux M., Schwessinger B., Albrecht C., Chinchilla D., Jones A., Holton N., Malinovsky F.G., Tör M., de Vries S., Zipfel C. (2011). The Arabidopsis Leucine-Rich Repeat Receptor-like Kinases BAK1/SERK3 and BKK1/SERK4 Are Required for Innate Immunity to Hemibiotrophic and Biotrophic Pathogens. Plant Cell.

[17] Nam K.H., Li J. (2002). BRI1/BAK1, a Receptor Kinase Pair Mediating Brassinosteroid Signaling. Cell.

[18] Liebrand T.W.H., Van Den Berg G.C.M., Zhang Z., Smit P., Cordewener J.H.G., America A.H.P., Sklenar J., Jones A.M.E., Tameling W.I.L., Robatzek S. (2013). Receptor-like Kinase SOBIR1/EVR Interacts with Receptor-like Proteins in Plant Immunity against Fungal Infection. Proc. Natl. Acad. Sci. USA.

[19] Ye Y., Ding Y., Jiang Q., Wang F., Sun J., Zhu C. (2017). The Role of Receptor-like Protein Kinases (RLKs) in Abiotic Stress Response in Plants. Plant Cell Rep..

[20] Ma X., Xu G., He P., Shan L. (2016). SERKing Coreceptors for Receptors. Trends Plant Sci..

[21] Diévart A., Clark S.E. (2003). Using Mutant Alleles to Determine the Structure and Function of Leucine-Rich Repeat Receptor-like Kinases. Curr. Opin. Plant Biol..

[22] Xi L., Wu X.N., Gilbert M., Schulze W.X. (2019). Classification and Interactions of LRR Receptors and Co-Receptors within the Arabidopsis Plasma Membrane—An Overview. Front. Plant Sci..

[23] Zhang H., Lin X., Han Z., Wang J., Qu L.J., Chai J. (2016). SERK Family Receptor-like Kinases Function as Co-Receptors with PXY for Plant Vascular Development. Mol. Plant.

[24] Hohmann U., Nicolet J., Moretti A., Hothorn L.A., Hothorn M. (2018). The SERK3 Elongated Allele Defines a Role for BIR Ectodomains in Brassinosteroid Signalling. Nat. Plants.

[25] Hohmann U., Santiago J., Nicolet J., Olsson V., Spiga F.M., Hothorn L.A., Butenko M.A., Hothorn M. (2018). Mechanistic Basis for the Activation of Plant Membrane Receptor Kinases by SERK-Family Coreceptors. Proc. Natl. Acad. Sci. USA.

[26] Chinchilla D., Shan L., He P., de Vries S., Kemmerling B. (2009). One for All: The Receptor-Associated Kinase BAK1. Trends Plant Sci..

[27] Postel S., Küfner I., Beuter C., Mazzotta S., Schwedt A., Borlotti A., Halter T., Kemmerling B., Nürnberger T. (2010). The Multifunctional Leucine-Rich Repeat Receptor Kinase BAK1 Is Implicated in Arabidopsis Development and Immunity. Eur. J. Cell Biol..

[28] Lin W., Li B., Lu D., Chen S., Zhu N., He P., Shan L. (2014). Tyrosine Phosphorylation of Protein Kinase Complex BAK1/BIK1 Mediates Arabidopsis Innate Immunity. Proc. Natl. Acad. Sci. USA.

[29] Jordá L., Sopeña-Torres S., Escudero V., Nuñez-Corcuera B., Delgado-Cerezo M., Torii K.U., Molina A. (2016). ERECTA and BAK1 Receptor like Kinases Interact to Regulate Immune Responses in Arabidopsis. Front. Plant Sci..

[30] Liu Y., Huang X., Li M., He P., Zhang Y. (2016). Loss-of-Function of Arabidopsis Receptor-like Kinase BIR1 Activates Cell Death and Defense Responses Mediated by BAK1 and SOBIR1. New Phytol..

[31] Wang Z., Meng P., Zhang X., Ren D., Yang S. (2011). BON1 Interacts with the Protein Kinases BIR1 and BAK1 in Modulation of Temperature-Dependent Plant Growth and Cell Death in Arabidopsis. Plant J..

[32] Bleckmann A., Weidtkamp-Peters S., Seidel C.A.M., Simon R. (2010). Stem Cell Signaling in Arabidopsis Requires CRN to Localize CLV2 to the Plasma Membrane. Plant Physiol..

[33] Cui Y., Hu C., Zhu Y., Cheng K., Li X., Wei Z., Xue L., Lin F., Shi H., Yi J. (2018). CIK Receptor Kinases Determine Cell Fate Specificatioduring Early Anther Development in Arabidopsis. Plant Cell.

[34] Liu P.L., Du L., Huang Y., Gao S.M., Yu M. (2017). Origin and Diversification of Leucine-Rich Repeat Receptor-like Protein Kinase (LRR-RLK) Genes in Plants. BMC Evol. Biol..

[35] He X., Feng T., Zhang D., Zhuo R., Liu M. (2019). Identification and Comprehensive Analysis of the Characteristics and Roles of Leucine-Rich Repeat Receptor-like Protein Kinase (LRR-RLK) Genes in *Sedum alfredii* Hance Responding to Cadmium Stress. Ecotoxicol. Environ. Saf..

[36] Liu P.L., Xie L.L., Li P.W., Mao J.F., Liu H., Gao S.M., Shi P.H., Gong J.Q. (2016). Duplication and Divergence of Leucine-Rich Repeat Receptor-like Protein Kinase (LRR-RLK) Genes in Basal *Angiosperm Amborella trichopoda*. Front. Plant Sci..

[37] Dufayard J.F., Bettembourg M., Fischer I., Droc G., Guiderdoni E., Périn C., Chantret N., Diévart A. (2017). New Insights on Leucine-Rich Repeats Receptor-like Kinase Orthologous Relationships in Angiosperms. Front. Plant Sci..

[38] Xiao Y., Xia W., Yang Y., Mason A.S., Lei X., Ma Z. (2013). Characterization and Evolution of Conserved MicroRNA through Duplication Events in Date Palm (*Phoenix dactylifera*). PLoS ONE.

[39] Huang S., Li R., Zhang Z., Li L., Gu X., Fan W., Lucas W.J., Wang X., Xie B., Ni P. (2009). The Genome of the Cucumber, *Cucumis sativus*, L.. Nat. Genet..

[40] Sun J., Li L., Wang P., Zhang S., Wu J. (2017). Genome-Wide Characterization, Evolution, and Expression Analysis of the Leucine-Rich Repeat Receptor-like Protein Kinase (LRR-RLK) Gene Family in Rosaceae Genomes. BMC Genom..

[41] Meng J., Yang J., Peng M., Liu X., He H. (2020). Genome-Wide Characterization, Evolution, and Expression Analysis of the Leucine-Rich Repeat Receptor-like Protein Kinase (Lrr-Rlk) Gene Family in *Medicago truncatula*. Life.

[42] Zhuang J., Peng R.H., Cheng Z.M., Zhang J., Cai B., Zhang Z., Gao F., Zhu B., Fu X.Y., Jin X.F. (2009). Genome-Wide Analysis of the Putative AP2/ERF Family Genes in *Vitis vinifera*. Sci. Hortic..

[43] Wu Y., Xun Q., Guo Y., Zhang J., Cheng K., Shi T., He K., Hou S., Gou X., Li J. (2016). Genome-Wide Expression Pattern Analyses of the Arabidopsis Leucine-Rich Repeat Receptor-like Kinases. Mol. Plant.

[44] Hirakawa H., Tsuchimoto S., Sakai H., Nakayama S., Fujishiro T., Kishida Y., Kohara M., Watanabe A., Yamada M., Aizu T. (2012). Upgraded Genomic Information of *Jatropha curcas* L.. Plant Biotechnol..

[45] Zhu H., Wang Y., Yin H., Gao M., Zhang Q., Chen Y. (2015). Genome-Wide Identification and Characterization of the LRR-RLK Gene Family in Two *Vernicia* Species. Int. J. Genom..

[46] Cao Y., Mo W., Li Y., Li W., Dong X., Liu M., Jiang L., Zhang L. (2021). Deciphering the Roles of Leucine-Rich Repeat Receptor-like Protein Kinases (LRR-RLKs) in Response to *Fusarium* Wilt in the *Vernicia fordii* (Tung Tree). Phytochemistry.

[47] Sakamoto T., Deguchi M., Brustolini O.J.B., Santos A.A., Silva F.F., Fontes E.P.B. (2012). The Tomato RLK Superfamily: Phylogeny and Functional Predictions about the Role of the LRRII-RLK Subfamily in Antiviral Defense. BMC Plant Biol..

[48] Shen S., Zhang Q., Shi Y., Sun Z., Zhang Q., Hou S., Wu R., Jiang L., Zhao X., Guo Y. (2020). Genome-Wide Analysis of the NAC Domain Transcription Factor Gene Family in *Theobroma cacao*. Genes.

[49] Song W., Wang B., Li X., Wei J., Chen L., Zhang D., Zhang W., Li R. (2015). Identification of Immune Related LRR-Containing Genes in Maize (*Zea mays* L.) by Genome-Wide Sequence Analysis. Int. J. Genom..

[50] Dezhsetan S. (2017). Genome Scanning for Identification and Mapping of Receptor-like Kinase (RLK) Gene Superfamily in *Solanum tuberosum*. Physiol. Mol. Biol. Plants.

[51] Wang J., Hu T., Wang W., Hu H., Wei Q., Bao C. (2019). Investigation of Evolutionary and Expressional Relationships in the Function of the Leucine-Rich Repeat Receptor-like Protein Kinase Gene Family (LRR-RLK) in the Radish (*Raphanus sativus* L.). Sci. Rep..

[52] Sun R., Wang S., Ma D., Liu C. (2018). Genome-Wide Analysis of LRR-RLK Gene Family in Four *Gossypium* Species and Expression Analysis during Cotton Development and Stress Responses. Genes.

[53] Magalhães D.M., Scholte L.L.S., Silva N.V., Oliveira G.C., Zipfel C., Takita M.A., De Souza A.A. (2016). LRR-RLK Family from Two *Citrus* Species: Genome-Wide Identification and Evolutionary Aspects. BMC Genom..

[54] Rameneni J.J., Lee Y., Dhandapani V., Yu X., Choi S.R., Oh M.H., Lim Y.P. (2015). Genomic and Post-Translational Modification Analysis of Leucine-Rich-Repeat Receptor-like Kinases in *Brassica rapa*. PLoS ONE.

[55] Li W., Chen M., Wang E., Hu L., Hawkesford M.J., Zhong L., Chen Z., Xu Z., Li L., Zhou Y. (2016). Genome-Wide Analysis of Autophagy-Associated Genes in Foxtail Millet (*Setaria italica* L.) and Characterization of the Function of SiATG8a in Conferring Tolerance to Nitrogen Starvation in Rice. BMC Genom..

[56] Truco M.J., Quiros C.F. (1994). Structure and Organization of the B Genome Based on a Linkage Map in *Brassica nigra*. Theor. Appl. Genet..

[57] Sun X., Wang G.L. (2011). Genome-Wide Identification, Characterization and Phylogenetic Analysis of the Rice LRR-Kinases. PLoS ONE.

[58] Liu Q., Chang S., Hartman G.L., Domier L.L. (2018). Assembly and Annotation of a Draft Genome Sequence for *Glycine latifolia*, a Perennial Wild Relative of Soybean. Plant J..

[59] Yang W., Yu M., Zou C., Lu C., Yu D., Cheng H., Jiang P., Feng X., Zhang Y., Wang Q. (2018). Genome-Wide Comparative Analysis of RNA-Binding Glycine-Rich Protein Family Genes between *Gossypium arboreum* and *Gossypium raimondii*. PLoS ONE.

[60] Cao Y., Han Y., Meng D., Li D., Jin Q., Lin Y., Cai Y. (2016). Structural, Evolutionary, and Functional Analysis of the Class III Peroxidase Gene Family in Chinese Pear (*Pyrus bretschneideri*). Front. Plant Sci..

[61] Zan Y., Ji Y., Zhang Y., Yang S., Song Y., Wang J. (2013). Genome-Wide Identification, Characterization and Expression Analysis of *Populus* Leucine-Rich Repeat Receptor-like Protein Kinase Genes. BMC Genom..

[62] Yang H., Bayer P.E., Tirnaz S., Edwards D., Batley J. (2021). Genome-Wide Identification and Evolution of Receptor-like Kinases (RLKs) and Receptor like Proteins (RLPs) in *Brassica juncea*. Biology.

[63] Zhou F., Guo Y., Qiu L.J. (2016). Genome-Wide Identification and Evolutionary Analysis of Leucine-Rich Repeat Receptor-like Protein Kinase Genes in Soybean. BMC Plant Biol..

[64] Shumayla, Sharma S., Kumar R., Mendu V., Singh K., Upadhyay S.K. (2016). Genomic Dissection and Expression Profiling Revealed Functional Divergence in *Triticum aestivum* Leucine Rich Repeat Receptor like Kinases (TaLRRKs). Front. Plant Sci..

[65] Wang X., Wu M.H., Xiao D., Huang R.L., Zhan J., Wang A.Q., He L.F. (2021). Genome-Wide Identification and Evolutionary Analysis of RLKs Involved in the Response to Aluminium Stress in Peanut. BMC Plant Biol..

[66] Mishra D., Suri G.S., Kaur G., Tiwari M. (2021). Comprehensive Analysis of Structural, Functional, and Evolutionary Dynamics of Leucine Rich Repeats-RLKs in *Thinopyrum elongatum*. Int. J. Biol. Macromol..

[67] Zoulias N., Harrison E.L., Casson S.A., Gray J.E. (2018). Molecular Control of Stomatal Development. Biochem. J..

[68] Zhu Y., Hu C., Cui Y., Zeng L., Li S., Zhu M., Meng F., Huang S., Long L., Yi J. (2021). Conserved and Differentiated Functions of CIK Receptor Kinases in Modulating Stem Cell Signaling in *Arabidopsis*. Mol. Plant.

[69] Ou Y., Kui H., Li J. (2021). Receptor-like Kinases in Root Development: Current Progress and Future Directions. Mol. Plant.

[70] Nadeau J.A. (2009). Stomatal Development: New Signals and Fate Determinants. Curr. Opin. Plant Biol..

[71] Cammarata J., Scanlon M.J. (2020). A Functionally Informed Evolutionary Framework for the Study of LRR-RLKs during Stem Cell Maintenance. J. Plant Res..

[72] Kim B.H., Kim S.Y., Nam K.H. (2013). Assessing the Diverse Functions of BAK1 and Its Homologs in *Arabidopsis*, beyond BR Signaling and PTI Responses. Mol. Cells.

[73] Shang Y., Dai C., Lee M.M., Kwak J.M., Nam K.H. (2016). BRI1-Associated Receptor Kinase 1 Regulates Guard Cell ABA Signaling Mediated by Open Stomata 1 in *Arabidopsis*. Mol. Plant.

[74] Kosaka A., Pastorczyk M., Piślewska-Bednarek M., Nishiuchi T., Ono E., Suemoto H., Ishikawa A., Frerigmann H., Kaido M., Mise K. (2021). Tryptophan-Derived Metabolites and BAK1 Separately Contribute to Arabidopsis Postinvasive Immunity against *Alternaria brassicicola*. Sci. Rep..

[75] Peng H.C., Kaloshian I. (2014). The Tomato Leucine-Rich Repeat Receptor-like Kinases SlSERK3A and SlSERK3B Have Overlapping Functions in Bacterial and Nematode Innate Immunity. PLoS ONE.

[76] Chaparro-Garcia A., Wilkinson R.C., Gimenez-Ibanez S., Findlay K., Coffey M.D., Zipfel C., Rathjen J.P., Kamoun S., Schornack S. (2011). The Receptor-like Kinase Serk3/Bak1 Is Required for Basal Resistance against the Late Blight Pathogen Phytophthora Infestans in *Nicotiana benthamiana*. PLoS ONE.

[77] Chen X., Zuo S., Schwessinger B., Chern M., Canlas P.E., Ruan D., Zhou X., Wang J., Daudi A., Petzold C.J. (2014). An XA21-Associated Kinase (OsSERK2) Regulates Immunity Mediated by the XA21 and XA3 Immune Receptors. Mol. Plant.

[78] Hu H., Xiong L., Yang Y. (2005). Rice SERK1 Gene Positively Regulates Somatic Embryogenesis of Cultured Cell and Host Defense Response against Fungal Infection. Planta.

[79] Derbyshire M., Mbengue M., Barascud M., Navaud O., Raffaele S. (2019). Small RNAs from the Plant Pathogenic Fungus *Sclerotinia sclerotiorum* Highlight Host Candidate Genes Associated with Quantitative Disease Resistance. Mol. Plant Pathol..

[80] Dong N., Yin W., Liu D., Zhang X., Yu Z., Huang W., Liu J., Yang Y., Meng W., Niu M. (2020). Regulation of Brassinosteroid Signaling and Salt Resistance by SERK2 and Potential Utilization for Crop Improvement in Rice. Front. Plant Sci..

[81] Li X., Ahmad S., Ali A., Guo C., Li H., Yu J., Zhang Y., Gao X., Guo Y. (2019). Characterization of Somatic Embryogenesis Receptor-like Kinase 4 as a Negative Regulator of Leaf Senescence in *Arabidopsis*. Cells.

[82] Wang X., Li X., Meisenhelder J., Hunter T., Yoshida S., Asami T., Chory J. (2005). Autoregulation and Homodimerization Are Involved in the Activation of the Plant Steroid Receptor BRI1. Dev. Cell.

[83] Goddard R., Peraldi A., Ridout C., Nicholson P. (2014). Enhanced Disease Resistance Caused by BRI1 Mutation Is Conserved between *Brachypodium distachyon* and Barley (*Hordeum vulgare*). Mol. Plant-Microbe Interact..

[84] Clouse S.D., Langford M., McMorris T.C. (1996). A Brassinosteroid-Insensitive Mutant in *Arabidopsis thaliana* Exhibits Multiple Defects in Growth and Development. Plant Physiol..

[85] Li J., Lease K.A., Tax F.E., Walker J.C. (2001). BRS1, a Serine Carboxypeptidase, Regulates BRI1 Signaling in *Arabidopsis thaliana*. Proc. Natl. Acad. Sci. USA.

[86] Feng Y., Yin Y., Fei S. (2015). Down-Regulation of BdBRI1, a Putative Brassinosteroid Receptor Gene Produces a Dwarf Phenotype with Enhanced Drought Tolerance in *Brachypodium distachyon*. Plant Sci..

[87] Holton N., Harrison K., Yokota T., Bishop G.J. (2008). Tomato BRI1 and Systemin Wound Signalling. Plant Signal. Behav..

[88] Huang S., Wang H., Gan S., Hussein M.A.M., Wang Q., Wang X., Zhang Y., Wang X. (2018). Molecular Identification and Functional Analysis of Brbri1 as Brassinosteroid Receptor Gene in *Brassica rapa*. Pak. J. Bot..

[89] Sharma A., Matsuoka M., Tanaka H., Komatsu S. (2001). Antisense Inhibition of a BRI1 Receptor Reveals Additional Protein Kinase Signaling Components Downstream to the Perception of Brassinosteroids in Rice. FEBS Lett..

[90] Scheer J.M., Pearce G., Ryan C.A. (2003). Generation of Systemin Signaling in Tobacco by Transformation with the Tomato Systemin Receptor Kinase Gene. Proc. Natl. Acad. Sci. USA.

[91] Deng X.G., Zhu T., Zou L.J., Han X.Y., Zhou X., Xi D.H., Zhang D.W., Lin H.H. (2016). Orchestration of Hydrogen Peroxide and Nitric Oxide in Brassinosteroid-Mediated Systemic Virus Resistance in *Nicotiana benthamiana*. Plant J..

[92] Singh A., Breja P., Khurana J.P., Khurana P. (2016). Wheat Brassinosteroid-Insensitive1 (TaBRI1) Interacts with Members of TaSERK Gene Family and Cause Early Flowering and Seed Yield Enhancement in *Arabidopsis*. PLoS ONE.

[93] Nie S., Huang S., Wang S., Cheng D., Liu J., Lv S., Li Q., Wang X. (2017). Enhancing Brassinosteroid Signaling via Overexpression of Tomato (*Solanum lycopersicum*) SlBRI1 Improves Major Agronomic Traits. Front. Plant Sci..

[94] Huang S., Zheng C., Zhao Y., Li Q., Liu J., Deng R., Lei T., Wang S., Wang X. (2021). RNA Interference Knockdown of the Brassinosteroid Receptor BRI1 in Potato (*Solanum tuberosum* L.) Reveals Novel Functions for Brassinosteroid Signaling in Controlling Tuberization. Sci. Hortic..

[95] Costa A.T., Bravo J.P., Krause-Sakate R., Maia I.G. (2016). The Receptor-like Kinase SlSOBIR1 Is Differentially Modulated by Virus Infection but Its Overexpression in Tobacco Has No Significant Impact on Virus Accumulation. Plant Cell Rep..

[96] Huang W.R.H., Schol C., Villanueva S.L., Heidstra R., Joosten M.H.A.J. (2021). Knocking out SOBIR1 in *Nicotiana benthamiana* Abolishes Functionality of Transgenic Receptor-like Protein Cf-4. Plant Physiol..

[97] Zhou Y., Sun L., Wassan G.M., He X., Shaban M., Zhang L., Zhu L., Zhang X. (2019). GbSOBIR1 Confers Verticillium Wilt Resistance by Phosphorylating the Transcriptional Factor GbbHLH171 in *Gossypium barbadense*. Plant Biotechnol. J..

[98] Zipfel C., Kunze G., Chinchilla D., Caniard A., Jones J.D.G., Boller T., Felix G. (2006). Perception of the Bacterial PAMP EF-Tu by the Receptor EFR Restricts Agrobacterium-Mediated Transformation. Cell.

[99] Lacombe S., Rougon-Cardoso A., Sherwood E., Peeters N., Dahlbeck D., Van Esse H.P., Smoker M., Rallapalli G., Thomma B.P.H.J., Staskawicz B. (2010). Interfamily Transfer of a Plant Pattern-Recognition Receptor Confers Broad-Spectrum Bacterial Resistance. Nat. Biotechnol..

[100] Holton N., Nekrasov V., Ronald P.C., Zipfel C. (2015). The Phylogenetically-Related Pattern Recognition Receptors EFR and XA21 Recruit Similar Immune Signaling Components in Monocots and Dicots. PLoS Pathog..

[101] Piazza S., Campa M., Pompili V., Costa L.D., Salvagnin U., Nekrasov V., Zipfel C., Malnoy M. (2021). The Arabidopsis Pattern Recognition Receptor EFR Enhances Fire Blight Resistance in Apple. Hortic. Res..

[102] Pfeilmeier S., George J., Morel A., Roy S., Smoker M., Stransfeld L., Downie J.A., Peeters N., Malone J.G., Zipfel C. (2019). Expression of the *Arabidopsis thaliana* Immune Receptor EFR in *Medicago truncatula* Reduces Infection by a Root Pathogenic Bacterium, but Not Nitrogen-Fixing Rhizobial Symbiosis. Plant Biotechnol. J..

[103] Boschi F., Schvartzman C., Murchio S., Ferreira V., Siri M.I., Galván G.A., Smoker M., Stransfeld L., Zipfel C., Vilaró F.L. (2017). Enhanced Bacterial Wilt Resistance in Potato through Expression of Arabidopsis Efr and Introgression of Quantitative Resistance from *Solanum commersonii*. Front. Plant Sci..

[104] Mosher S., Seybold H., Rodriguez P., Stahl M., Davies K.A., Dayaratne S., Morillo S.A., Wierzba M., Favery B., Keller H. (2013). The Tyrosine-Sulfated Peptide Receptors PSKR1 and PSY1R Modify the Immunity of Arabidopsis to Biotrophic and Necrotrophic Pathogens in an Antagonistic Manner. Plant J..

[105] Yang W., Zhang B., Qi G., Shang L., Liu H., Ding X., Chu Z. (2019). Identification of the Phytosulfokine Receptor 1 (OsPSKR1) Confers Resistance to Bacterial Leaf Streak in Rice. Planta.

[106] Nakaminami K., Okamoto M., Higuchi-Takeuchi M., Yoshizumi T., Yamaguchi Y., Fukao Y., Shimizu M., Ohashi C., Tanaka M., Matsui M. (2018). AtPep3 Is a Hormone-like Peptide That Plays a Role in the Salinity Stress Tolerance of Plants. Proc. Natl. Acad. Sci. USA.

[107] Zheng X., Kang S., Jing Y., Ren Z., Li L., Zhou J.M., Berkowitz G., Shi J., Fu A., Lan W. (2018). Danger-Associated Peptides Close Stomata by OST1-Independent Activation of Anion Channels in Guard Cells. Plant Cell.

[108] Xu S., Liao C.J., Jaiswal N., Lee S., Yun D.J., Lee S.Y., Garvey M., Kaplan I., Mengiste T. (2018). Tomato PEPR1 ORTHOLOG RECEPTOR-LIKE KINASE1 Regulates Responses to Systemin, Necrotrophic Fungi, and Insect Herbivory. Plant Cell.

[109] Shang Y., Yang D., Ha Y., Nam K.H. (2021). BAK1-Induced RPK1 Phosphorylation Is Essential for RPK1-Mediated Cell Death in *Arabidopsis*. Biochem. Biophys. Res. Commun..

[110] Choi D.S., Hwang I.S., Hwang B.K. (2012). Requirement of the Cytosolic Interaction between PATHOGENESIS-RELATED PROTEIN10 and LEUCINE-RICH REPEAT PROTEIN1 for Cell Death and Defense Signaling in Pepper. Plant Cell.

[111] Chen X., Wang T., Rehman A.U., Wang Y., Qi J., Li Z., Song C., Wang B., Yang S., Gong Z. (2021). Arabidopsis U-Box E3 Ubiquitin Ligase PUB11 Negatively Regulates Drought Tolerance by Degrading the Receptor-like Protein Kinases LRR1 and KIN7. J. Integr. Plant Biol..

[112] Isner J.C., Begum A., Nuehse T., Hetherington A.M., Maathuis F.J.M. (2018). KIN7 Kinase Regulates the Vacuolar TPK1 K+ Channel during Stomatal Closure. Curr. Biol..

[113] Thapa G., Gunupuru L.R., Hehir J.G., Kahla A., Mullins E., Doohan F.M. (2018). A Pathogen-Responsive Leucine Rich Receptor like Kinase Contributes to Fusarium Resistance in Cereals. Front. Plant Sci..

[114] Caddell D.F., Park C.J., Thomas N.C., Canlas P.E., Ronald P.C. (2017). Silencing of the Rice Gene LRR1 Compromises Rice Xa21 Transcript Accumulation and XA21-Mediated Immunity. Rice.

[115] ten Hove C.A., de Jong M., Lapin D., Andel A., Sanchez-Perez G.F., Tarutani Y., Suzuki Y., Heidstra R., van den Ackerveken G. (2011). Trans-Repression of Gene Activity Upstream of T-DNA Tagged Rlk902 Links Arabidopsis Root Growth Inhibition and Downy Mildew Resistance. PLoS ONE.

[116] Zhao Y., Wu G., Shi H., Tang D. (2019). RECEPTOR-LIKE KINASE 902 Associates with and Phosphorylates BRASSINOSTEROID-SIGNALING KINASE1 to Regulate Plant Immunity. Mol. Plant.

[117] Fontes E.P.B., Santos A.A., Luz D.F., Waclawovsky A.J., Chory J. (2004). The Geminivirus Nuclear Shuttle Protein Is a Virulence Factor That Suppresses Transmembrane Receptor Kinase Activity. Genes Dev..

[118] Wang M., Weiberg A., Dellota E., Yamane D., Jin H. (2017). Botrytis Small RNA Bc-SiR37 Suppresses Plant Defense Genes by Cross-Kingdom RNAi. RNA Biol..

[119] Liu T., Jiang G.Q., Yao X.F., Liu C.M. (2021). The Leucine-Rich Repeat Receptor-like Kinase OsERL Plays a Critical Role in Anther Lobe Formation in Rice. Biochem. Biophys. Res. Commun..

[120] Nanda A.K., El Habti A., Hocart C.H., Masle J. (2019). ERECTA Receptor-Kinases Play a Key Role in the Appropriate Timing of Seed Germination under Changing Salinity. J. Exp. Bot..

[121] Yu Z., Zhang D., Xu Y., Jin S., Zhang L., Zhang S., Yang G., Huang J., Yan K., Wu C. (2019). CEPR2 Phosphorylates and Accelerates the Degradation of PYR/PYLs in *Arabidopsis*. J. Exp. Bot..

[122] Jung C.G., Hwang S.G., Park Y.C., Park H.M., Kim D.S., Park D.H., Jang C.S. (2015). Molecular Characterization of the Cold- and Heat-Induced Arabidopsis PXL1 Gene and Its Potential Role in Transduction Pathways under Temperature Fluctuations. J. Plant Physiol..

[123] Pfister A., Barberon M., Alassimone J., Kalmbach L., Lee Y., Vermeer J.E.M., Yamazaki M., Li G., Maurel C., Takano J. (2014). A Receptor-like Kinase Mutant with Absent Endodermal Diffusion Barrier Displays Selective Nutrient Homeostasis Defects. elife.

[124] Tunc-Ozdemir M., Jones A.M. (2017). BRL3 and AtRGS1 Cooperate to Fine Tune Growth Inhibition and ROS Activation. PLoS ONE.

[125] Fàbregas N., Lozano-Elena F., Blasco-Escámez D., Tohge T., Martínez-Andújar C., Albacete A., Osorio S., Bustamante M., Riechmann J.L., Nomura T. (2018). Overexpression of the Vascular Brassinosteroid Receptor BRL3 Confers Drought Resistance without Penalizing Plant Growth. Nat. Commun..

[126] Nakamura A., Fujioka S., Sunohara H., Kamiya N., Hong Z., Inukai Y., Miura K., Takatsuto S., Yoshida S., Ueguchi-Tanaka M. (2006). The Role of OsBRI1 and Its Homologous Genes, OsBRL1 and OsBRL3, in Rice. Plant Physiol..

[127] Pitorre D., Llauro C., Jobet E., Guilleminot J., Brizard J.P., Delseny M., Lasserre E. (2010). RLK7, a Leucine-Rich Repeat Receptor-like Kinase, Is Required for Proper Germination Speed and Tolerance to Oxidative Stress in *Arabidopsis thaliana*. Planta.

[128] Replogle A., Wang J., Paolillo V., Smeda J., Kinoshita A., Durbak A., Tax F.E., Wang X., Sawa S., Mitchum M.G. (2013). Synergistic Interaction of Clavata1, Clavata2, and Receptor-like Protein Kinase 2 in Cyst Nematode Parasitism of Arabidopsis. Mol. Plant-Microbe Interact..

[129] Hu C., Zhu Y., Cui Y., Cheng K., Liang W., Wei Z., Zhu M., Yin H., Zeng L., Xiao Y. (2018). A Group of Receptor Kinases Are Essential for CLAVATA Signalling to Maintain Stem Cell Homeostasis. Nat. Plants.

[130] Xu F., Meng T., Li P., Yu Y., Cui Y., Wang Y., Gong Q., Wang N.N. (2011). A Soybean Dual-Specificity Kinase, GmSARK, and Its Arabidopsis Homolog, AtSARK, Regulate Leaf Senescence through Synergistic Actions of Auxin and Ethylene. Plant Physiol..

[131] Li P., Yang H., Liu G., Ma W., Li C., Huo H., He J., Liu L. (2018). PpSARK Regulates Moss Senescence and Salt Tolerance through ABA Related Pathway. Int. J. Mol. Sci..

[132] da Silva H.A.P., Caetano V.S., Pessoa D.D.V., Pacheco R.S., Simoes-Araujo J.L. (2019). Molecular and Biochemical Changes of Aging-Induced Nodules Senescence in Common Bean. Symbiosis.

[133] Soltabayeva A., Ongaltay A., Omondi J.O., Srivastava S. (2021). Morphological, Physiological and Molecular Markers for Salt-Stressed Plants. Plants.

[134] Meng X., Zhou J., Tang J., Li B., de Oliveira M.V.V., Chai J., He P., Shan L. (2016). Ligand-Induced Receptor-like Kinase Complex Regulates Floral Organ Abscission in Arabidopsis. Cell Rep..

[135] Li Z., Wang Y., Huang J., Ahsan N., Biener G., Paprocki J., Thelen J.J., Raicu V., Zhao D. (2017). Two SERK Receptor-like Kinases Interact with EMS1 to Control Anther Cell Fate Determination. Plant Physiol..

[136] Lu D., Wu S., Gao X., Zhang Y., Shan L., He P. (2010). A Receptor-like Cytoplasmic Kinase, BIK1, Associates with a Flagellin Receptor Complex to Initiate Plant Innate Immunity. Proc. Natl. Acad. Sci. USA.

[137] Li L., Li M., Yu L., Zhou Z., Liang X., Liu Z., Cai G., Gao L., Zhang X., Wang Y. (2014). The FLS2-Associated Kinase BIK1 Directly Phosphorylates the NADPH Oxidase RbohD to Control Plant Immunity. Cell Host Microbe.

[138] Zhu Y., Wang Y., Li R., Song X., Wang Q., Huang S., Jin J.B., Liu C.M., Lin J. (2010). Analysis of Interactions among the CLAVATA3 Receptors Reveals a Direct Interaction between CLAVATA2 and CORYNE in Arabidopsis. Plant J..

[139] Schulze B., Mentzel T., Jehle A.K., Mueller K., Beeler S., Boller T., Felix G., Chinchilla D. (2010). Rapid Heteromerization and Phosphorylation of Ligand-Activated Plant Transmembrane Receptors and Their Associated Kinase BAK1. J. Biol. Chem..

[140] Amorim-Silva V., García-Moreno Á., Castillo A.G., Lakhssassi N., Del Valle A.E., Pérez-Sancho J., Li Y., Posé D., Pérez-Rodriguez J., Lin J. (2019). TTL Proteins Scaffold Brassinosteroid Signaling Components at the Plasma Membrane to Optimize Signal Transduction in Arabidopsis. Plant Cell.

[141] Santiago J., Henzler C., Hothorn M. (2013). Molecular Mechanism for Plant Steroid Receptor Activation by Somatic Embryogenesis Co-Receptor Kinases. Science.

[142] Nicaise V., Joe A., Jeong B.R., Korneli C., Boutrot F., Westedt I., Staiger D., Alfano J.R., Zipfel C. (2013). Pseudomonas HopU1 Modulates Plant Immune Receptor Levels by Blocking the Interaction of Their MRNAs with GRP7. EMBO J..

[143] Wang J., Li H., Han Z., Zhang H., Wang T., Lin G., Chang J., Yang W., Chai J. (2015). Allosteric Receptor Activation by the Plant Peptide Hormone Phytosulfokine. Nature.

[144] Kinoshita A., Betsuyaku S., Osakabe Y., Mizuno S., Nagawa S., Stahl Y., Simon R., Yamaguchi-Shinozaki K., Fukuda H., Sawa S. (2010). RPK2 Is an Essential Receptor-like Kinase That Transmits the CLV3 Signal in Arabidopsis. Development.

[145] Guo Y., Han L., Hymes M., Denver R., Clark S.E. (2010). CLAVATA2 Forms a Distinct CLE-Binding Receptor Complex Regulating Arabidopsis Stem Cell Specification. Plant J..

[146] He K., Gou X., Yuan T., Lin H., Asami T., Yoshida S., Russell S.D., Li J. (2007). BAK1 and BKK1 Regulate Brassinosteroid-Dependent Growth and Brassinosteroid-Independent Cell-Death Pathways. Curr. Biol..

[147] Meng X., Chen X., Mang H., Liu C., Yu X., Gao X., Torii K.U., He P., Shan L. (2015). Differential Function of Arabidopsis SERK Family Receptor-like Kinases in Stomatal Patterning. Curr. Biol..

[148] Mou S., Zhang X., Han Z., Wang J., Gong X., Chai J. (2017). CLE42 Binding Induces PXL2 Interaction with SERK2. Protein Cell.

[149] Wang X., Kota U., He K., Blackburn K., Li J., Goshe M.B., Huber S.C., Clouse S.D. (2008). Sequential Transphosphorylation of the BRI1/BAK1 Receptor Kinase Complex Impacts Early Events in Brassinosteroid Signaling. Dev. Cell.

[150] Franco-Orozco B., Berepiki A., Ruiz O., Gamble L., Griffe L.L., Wang S., Birch P.R.J., Kanyuka K., Avrova A. (2017). A New Proteinaceous Pathogen-Associated Molecular Pattern (PAMP) Identified in Ascomycete Fungi Induces Cell Death in Solanaceae. New Phytol..

[151] Crook A.D., Schnabel E.L., Frugoli J.A. (2016). The Systemic Nodule Number Regulation Kinase SUNN in *Medicago truncatula* Interacts with MtCLV2 and MtCRN. Plant J..

[152] Smakowska-Luzan E., Mott G.A., Parys K., Stegmann M., Howton T.C., Layeghifard M., Neuhold J., Lehner A., Kong J., Grünwald K. (2018). An Extracellular Network of Arabidopsis Leucine-Rich Repeat Receptor Kinases. Nature.

[153] Mott G.A., Smakowska-Luzan E., Pasha A., Parys K., Howton T.C., Neuhold J., Lehner A., Grünwald K., Stolt-Bergner P., Provart N.J. (2019). Data Descriptor: Map of Physical Interactions between Extracellular Domains of Arabidopsis Leucine-Rich Repeat Receptor Kinases. Sci. Data.

[154] Li B., Ferreira M.A., Huang M., Camargos L.F., Yu X., Teixeira R.M., Carpinetti P.A., Mendes G.C., Gouveia-Mageste B.C., Liu C. (2019). The Receptor-like Kinase NIK1 Targets FLS2/BAK1 Immune Complex and Inversely Modulates Antiviral and Antibacterial Immunity. Nat. Commun..

[155] Karlova R., Boeren S., Russinova E., Aker J., Vervoort J., De Vries S. (2006). The Arabidopsis SOMATIC EMBRYOGENESIS RECEPTOR-LIKE KINASE1 Protein Complex Includes BRASSINOSTEROID-INSENSITIVE1. Plant Cell.

[156] Sun Y., Li L., Macho A.P., Han Z., Hu Z., Zipfel C., Zhou J.-M., Chai J. (2013). Structural Basis for Flg22-Induced Activation of the Arabidopsis FLS2-BAK1 Immune Complex. Science.

[157] He Y., Zhou J., Shan L., Meng X. (2018). Plant Cell Surface Receptor-Mediated Signaling-A Common Theme amid Diversity. J. Cell Sci..

[158] Albrecht C., Russinova E., Hecht V., Baaijens E., de Vries S. (2005). The *Arabidopsis thaliana* SOMATIC EMBRYOGENESIS RECEPTOR-LIKE KINASES1 and 2 Control Male Sporogenesis. Plant Cell.

[159] Tang J., Han Z., Sun Y., Zhang H., Gong X., Chai J. (2015). Structural Basis for Recognition of an Endogenous Peptide by the Plant Receptor Kinase PEPR1. Cell Res..

[160] Xiang T., Zong N., Zou Y., Wu Y., Zhang J., Xing W., Li Y., Tang X., Zhu L., Chai J. (2008). Pseudomonas Syringae Effector AvrPto Blocks Innate Immunity by Targeting Receptor Kinases. Curr. Biol..

[161] Ranf S., Eschen-Lippold L., Pecher P., Lee J., Scheel D. (2011). Interplay between Calcium Signalling and Early Signalling Elements during Defence Responses to Microbe- or Damage-Associated Molecular Patterns. Plant J..

[162] Kim S.Y., Warpeha K.M., Huber S.C. (2019). The Brassinosteroid Receptor Kinase, BRI1, Plays a Role in Seed Germination and the Release of Dormancy by Cold Stratification. J. Plant Physiol..

[163] Kemmerling B., Halter T., Mazzotta S., Mosher S., Nürnberger T. (2011). A Genome-Wide Survey for Arabidopsis Leucine-Rich Repeat Receptor Kinases Implicated in Plant Immunity. Front. Plant Sci..

[164] Wang X., Jiang N., Liu J., Liu W., Wang G.L. (2014). The Role of Effectors and Host Immunity in Plant–Necrotrophic Fungal Interactions. Virulence.

[165] Loivamäki M., Stührwohldt N., Deeken R., Steffens B., Roitsch T., Hedrich R., Sauter M. (2010). A Role for PSK Signaling in Wounding and Microbial Interactions in Arabidopsis. Physiol. Plant..

[166] Furukawa K., Hoshi Y., Maeda T., Nakajima T., Abe K. (2005). Aspergillus Nidulans HOG Pathway Is Activated Only by Two-Component Signalling Pathway in Response to Osmotic Stress. Mol. Microbiol..

[167] Carvalho C.M., Santos A.A., Pires S.R., Rocha C.S., Saraiva D.I., Machado J.P.B., Mattos E.C., Fietto L.G., Fontes E.P.B. (2008). Regulated Nuclear Trafficking of RpL10A Mediated by NIK1 Represents a Defense Strategy of Plant Cells against Virus. PLoS Pathog..

[168] Shang Y., Yang D., Ha Y., Shin H.Y., Nam K.H. (2020). Receptor-like Protein Kinases RPK1 and BAK1 Sequentially Form Complexes with the Cytoplasmic Kinase OST1 to Regulate ABA-Induced Stomatal Closure. J. Exp. Bot..

[169] Osakabe Y., Mizuno S., Tanaka H., Maruyama K., Osakabe K., Todaka D., Fujita Y., Kobayashi M., Shinozaki K., Yamaguchi-Shinozaki K. (2010). Overproduction of the Membrane-Bound Receptor-like Protein Kinase 1, RPK1, Enhances Abiotic Stress Tolerance in Arabidopsis. J. Biol. Chem..

[170] Schoonbeek H.J., Wang H.H., Stefanato F.L., Craze M., Bowden S., Wallington E., Zipfel C., Ridout C.J. (2015). Arabidopsis EF-Tu Receptor Enhances Bacterial Disease Resistance in Transgenic Wheat. New Phytol..

[171] Shen H., Zhong X., Zhao F., Wang Y., Yan B., Li Q., Chen G., Mao B., Wang J., Li Y. (2015). Overexpression of Receptor-like Kinase ERECTA Improves Thermotolerance in Rice and Tomato. Nat. Biotechnol..

[172] Sakai K., Citerne S., Antelme S., Le Bris P., Daniel S., Bouder A., D’Orlando A., Cartwright A., Tellier F., Pateyron S. (2021). BdERECTA Controls Vasculature Patterning and Phloem-Xylem Organization in Brachypodium Distachyon. BMC Plant Biol..

[173] Zhang Y., Li S., Xue S., Yang S., Huang J., Wang L. (2018). Phylogenetic and CRISPR/Cas9 Studies in Deciphering the Evolutionary Trajectory and Phenotypic Impacts of Rice ERECTA Genes. Front. Plant Sci..

[174] De Gruijl F.R., Rebel H. (2008). Early Events in UV Carcinogenesis-DNA Damage, Target Cells and Mutant P53 Foci. Photochem. Photobiol..

[175] Jaiswal N., Liao C.J., Mengesha B., Han H., Lee S., Sharon A., Zhou Y., Mengiste T. (2022). Regulation of Plant Immunity and Growth by Tomato Receptor-like Cytoplasmic Kinase TRK1. New Phytol..

[176] Araya T., Miyamoto M., Wibowo J., Suzuki A., Kojima S., Tsuchiya Y.N., Sawa S., Fukuda H., Von Wirén N., Takahashi H. (2014). CLE-CLAVATA1 Peptide-Receptor Signaling Module Regulates the Expansion of Plant Root Systems in a Nitrogen-Dependent Manner. Proc. Natl. Acad. Sci. USA.

[177] Wang L., Xu Y.Y., Li J., Powell R.A., Xu Z.H., Chong K. (2007). Transgenic Rice Plants Ectopically Expressing AtBAK1 Are Semi-Dwarfed and Hypersensitive to 24-Epibrassinolide. J. Plant Physiol..

[178] Hu L., Ye M., Kuai P., Ye M., Erb M., Lou Y. (2018). OsLRR-RLK1, an Early Responsive Leucine-Rich Repeat Receptor-like Kinase, Initiates Rice Defense Responses against a Chewing Herbivore. New Phytol..

[179] Brotman Y., Landau U., Pnini S., Lisec J., Balazadeh S., Mueller-Roeber B., Zilberstein A., Willmitzer L., Chet I., Viterbo A. (2012). The LysM Receptor-like Kinase LysM RLK1 Is Required to Activate Defense and Abiotic-Stress Responses Induced by Overexpression of Fungal Chitinases in Arabidopsis Plants. Mol. Plant.

[180] Qu X., Cao B., Kang J., Wang X., Han X., Jiang W., Shi X., Zhang L., Cui L., Hu Z. (2019). Fine-Tuning Stomatal Movement through Small Signaling Peptides. Front. Plant Sci..

[181] Ali S.S., Gunupuru L.R., Kumar G.B.S., Khan M., Scofield S., Nicholson P., Doohan F.M. (2014). Plant Disease Resistance Is Augmented in Uzu Barley Lines Modified in the Brassinosteroid Receptor BRI1. BMC Plant Biol..

