# Supplementary material for: Receptor-like Kinases (LRR-RLKs) in Response of Plants to Biotic and Abiotic Stresses
Source: Plants (Basel). 2022 Oct 10;11(19):2660. doi: 10.3390/plants11192660 (PMC9572924; doi:10.3390/plants11192660)
Supplement: Supplementary file 1 [file plants-11-02660-s001.zip › plants-1881702-Supplementary materials.pdf]

plants

MDPI

Supplementary Materials

Receptor-like Kinases (LRR-RLKs) in Response of Plants to Biotic and Abiotic Stresses

Aigerim Soltabayeva [1,*], Nurbanu Dauletova [1], Symbat Serik [1], Margulan Sandybek [1], John Okoth Omondi [2], Assylay Kurmanbayeva [3] and Sudhakar Srivastava [4]

[1] Biology Department, School of Science and Humanities, Nazarbayev University, Astana 010000, Kazakhstan
[2] International Institute of Tropical Agriculture, Lilongwe P.O. Box 30258, Malawi
[3] Department of Biotechnology and Microbiology, L.N. Gumilyov Eurasian National University, Astana 010000, Kazakhstan
[4] NCS-TCP, National Institute of Plant Genome Research, New Delhi 110067, India

* Correspondence: aigerim.soltabayeva@nu.edu.kz.

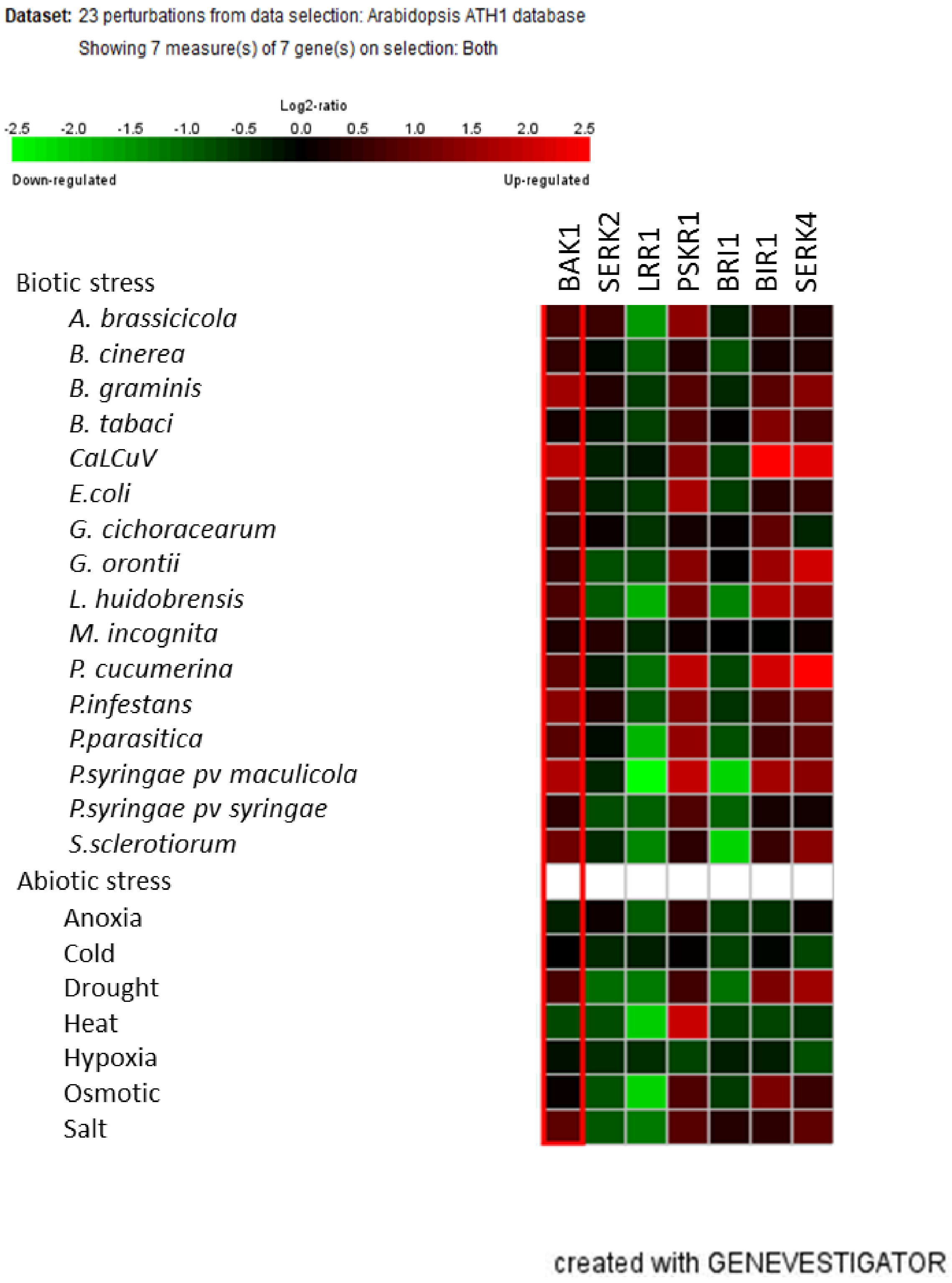

Supplementary Figure S1. Microarray analysis of the biotic and abiotic stress related LRR-RLK genes transcripts in response to biotic and abiotic stresses in WT (Col ecotype). Data was obtained from published microarray collections from Affymetrix *Arabidopsis* ATH1 Genome Array platform in Genevestigator (https://genevestigator.com/). Compendium-wide analysis was used to display the results by a condition search tool called perturbations. Data is displayed in the form of heatmap, which is based on Log2-ratio. Biotic and abiotic stress related LRR-RLK genes list from Supplemental table S1.

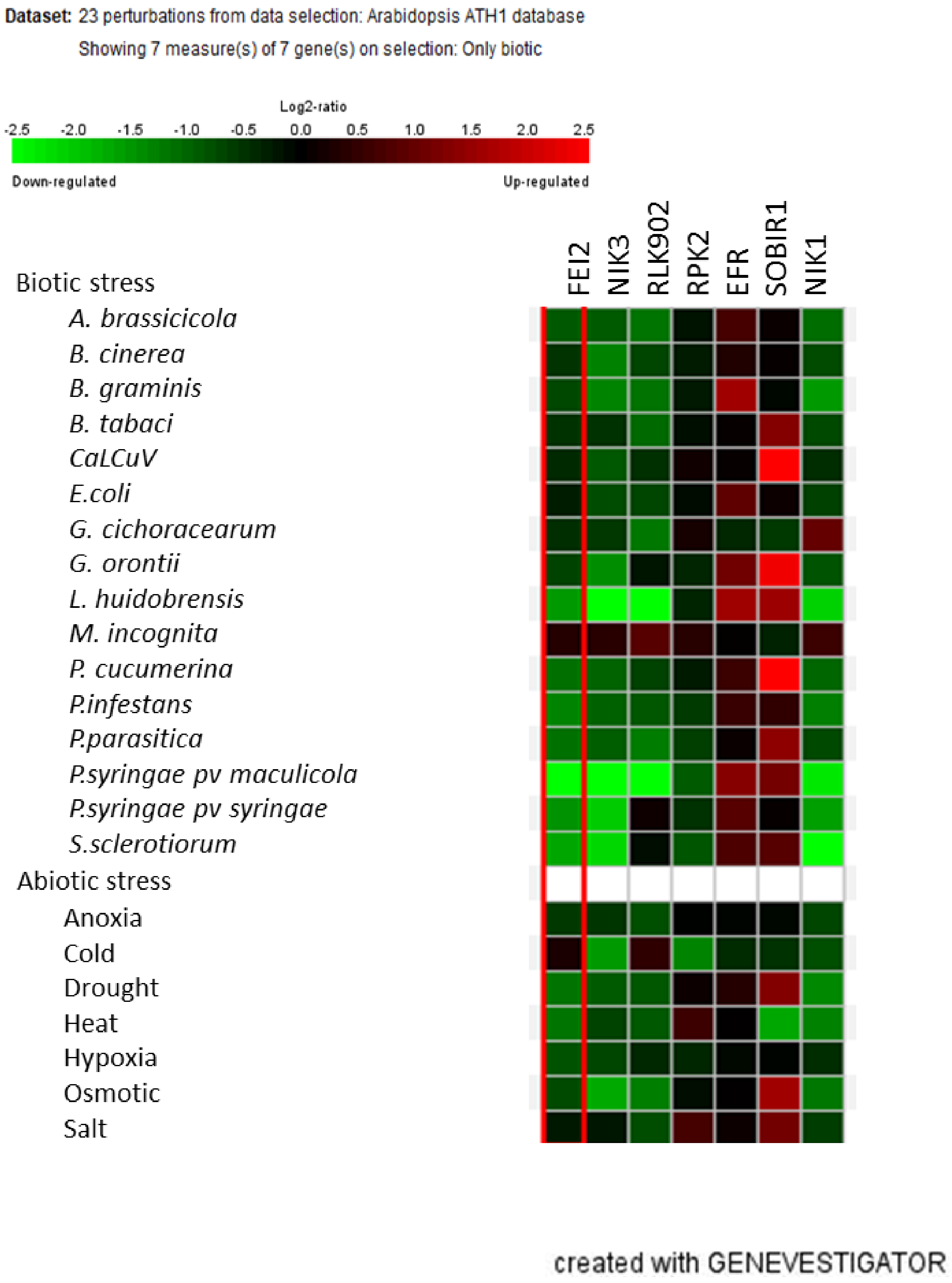

Supplementary Figure S2. Microarray analysis of the biotic stress related LRR-RLK genes transcripts in response to biotic and abiotic stresses in WT (Col ecotype). Data was obtained from published microarray collections from Affymetrix *Arabidopsis* ATH1 Genome Array platform in Genevestigator (https://genevestigator.com/). Compendium-wide analysis was used to display the results by a condition search tool called perturbations. Data is displayed in the form of heatmap, which is based on Log2-ratio. Biotic stress related LRR-RLK genes list from Supplemental table 1.

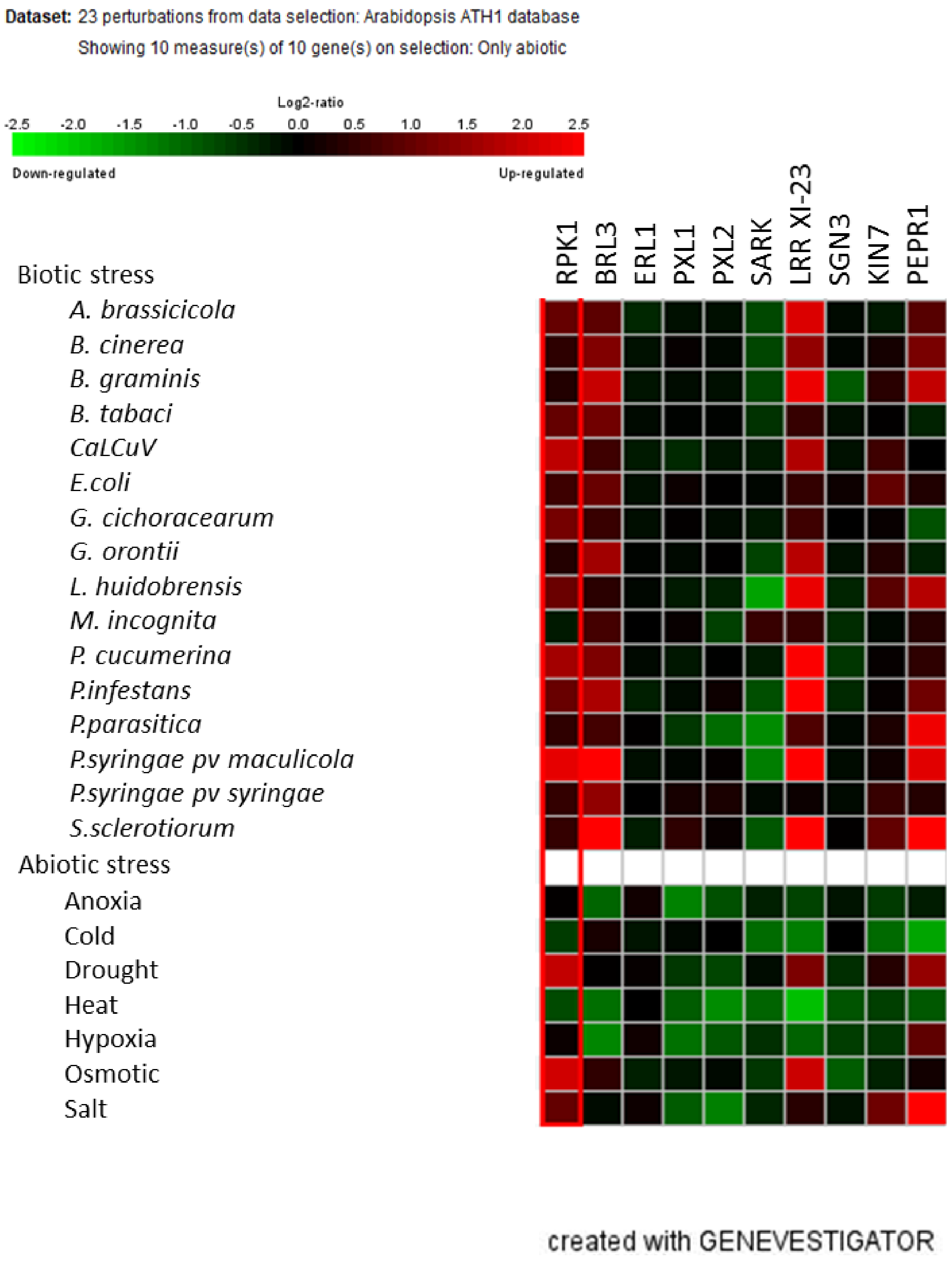

Supplementary Figure S3. Microarray analysis of the abiotic stress related LRR-RLK genes transcripts in response to biotic and abiotic stresses in WT (Col ecotype). Data was obtained from published microarray collections from Affymetrix *Arabidopsis* ATH1 Genome Array platform in Genevestigator (https://genevestigator.com/). Compendium-wide analysis was used to display the results by a condition search tool called perturbations. Data is displayed in the form of heatmap, which is based on Log2-ratio. Abiotic stress related LRR-RLK genes list from Supplemental table 1.

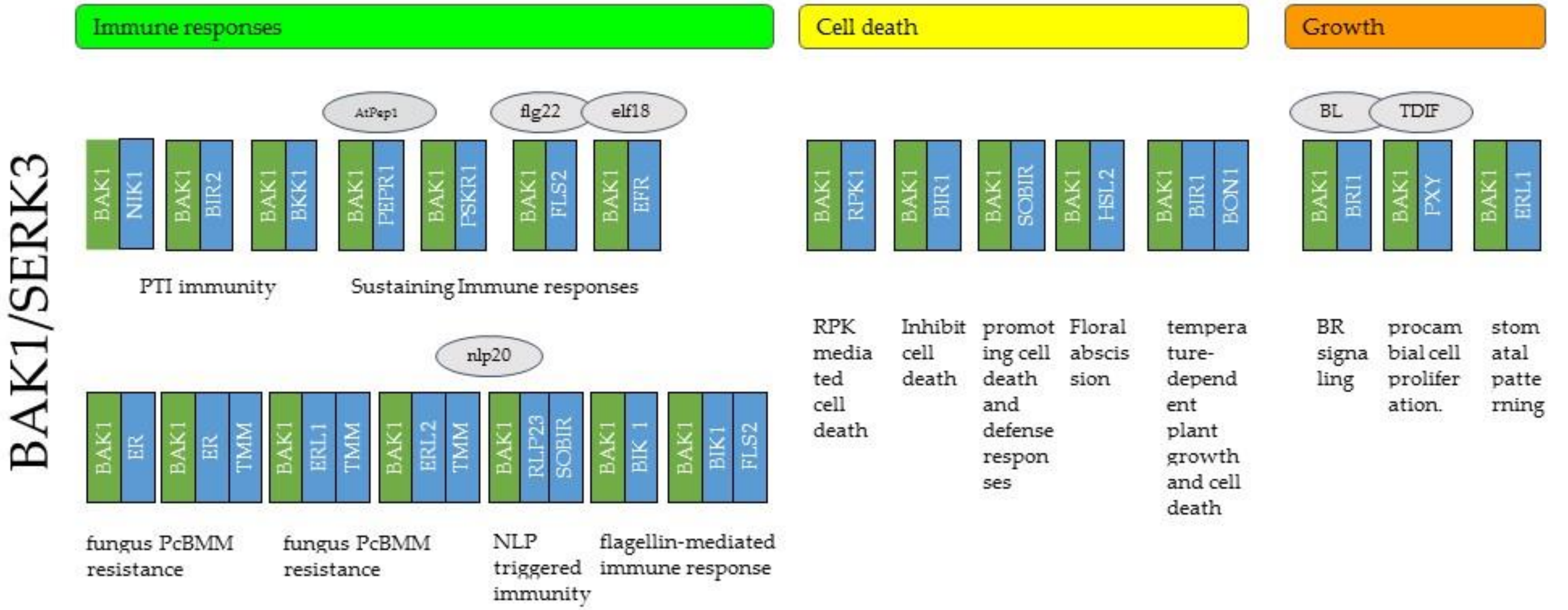

Supplementary Figure S4. Protein interactions of BAK1/**SERK3** with other LRR-RLK with experimental proved functional role.

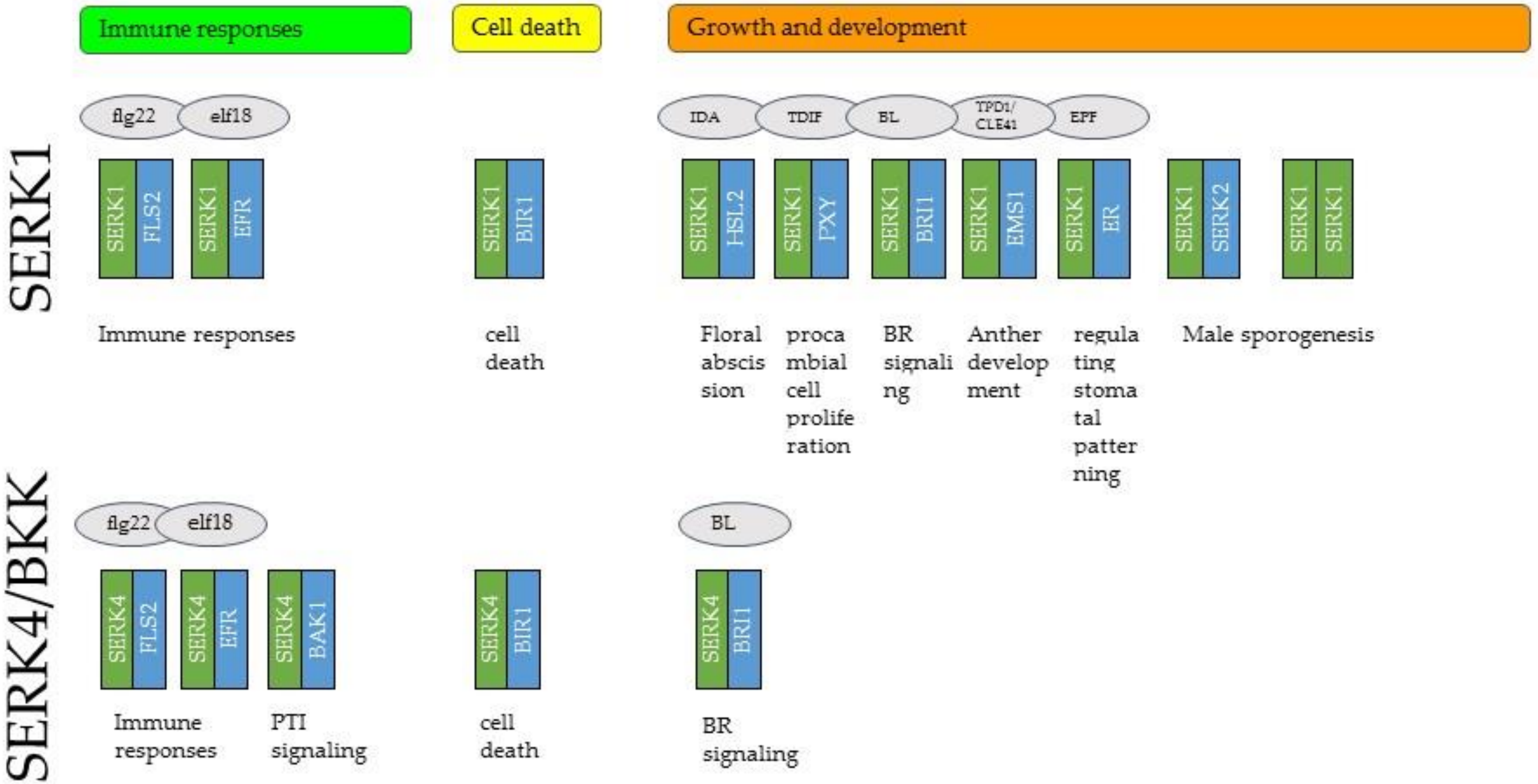

Supplementary Figure S5. Protein interactions of **SERK1 and SERK4** with other LRR-RLK with experimental proved functional role.

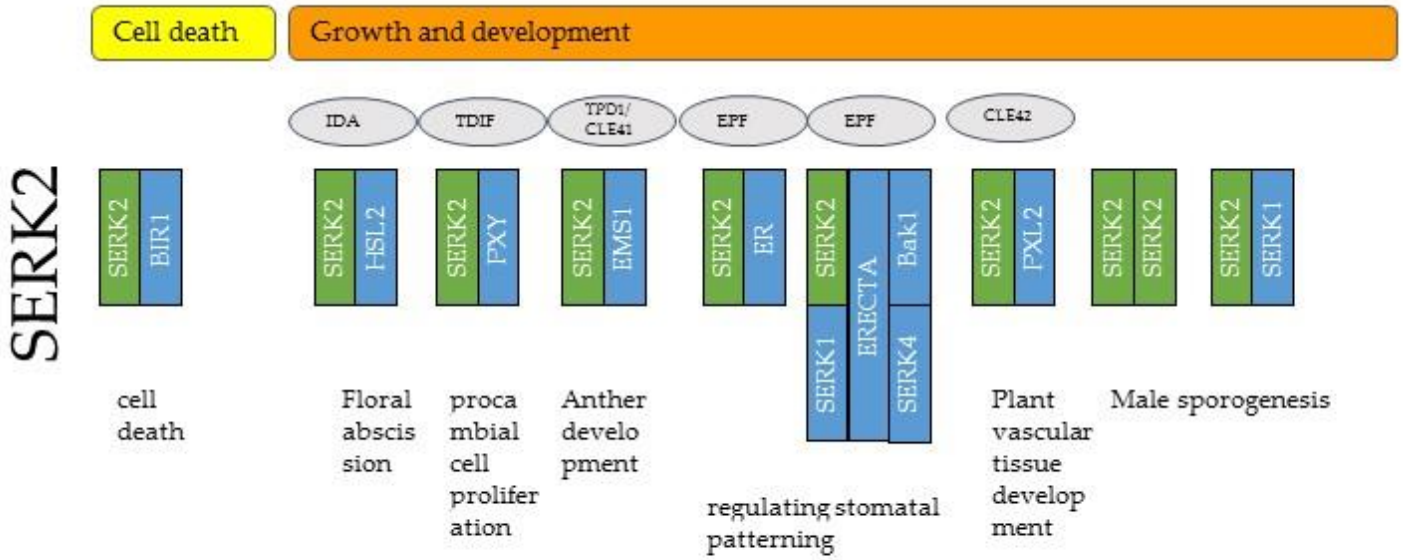

Supplementary Figure S6. Protein interactions of **SERK2** with other LRR-RLK with experimental proved functional role.

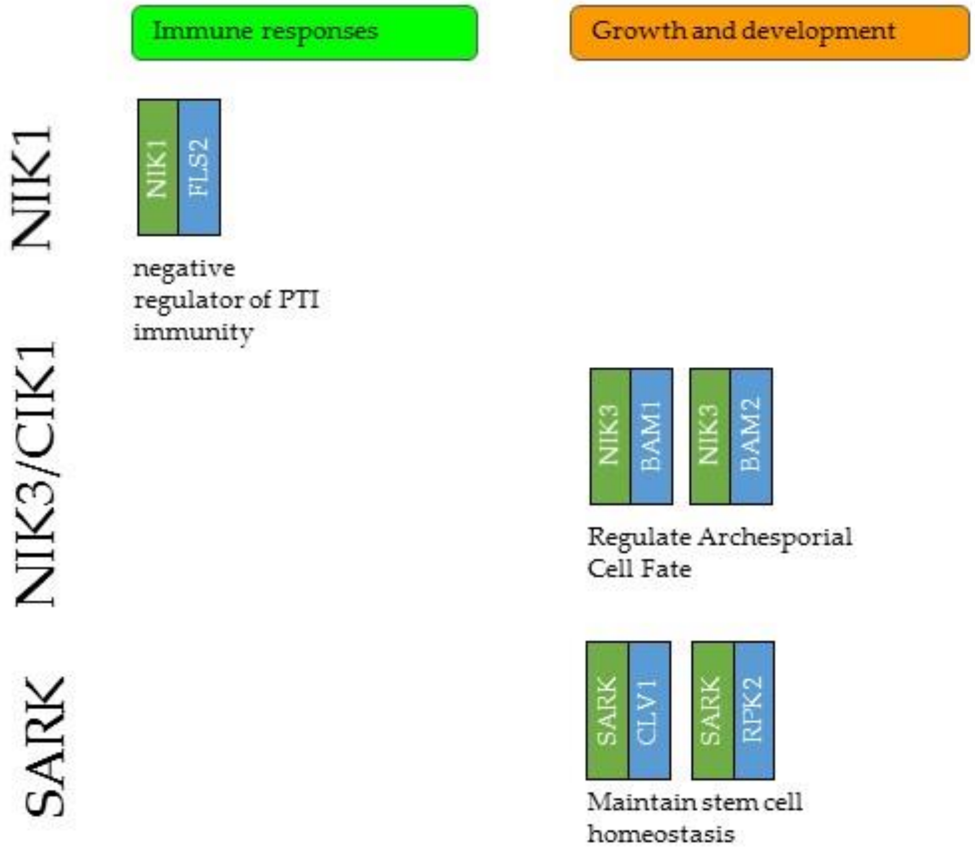

Supplementary Figure S7. Protein interactions of **CIK's (NIK1, NIK3 and SARK)** with other LRR-RLK with experimental proved functional role.

Table S1. Phenotypes of single mutants of LRR-RLK genes in *A.thaliana* tested under different biotic and abiotic stresses. The LRR-RLK genes names and their numbers were given. All data taken from different research studies about LRR-RLK genes investigating them under biotic (organism/s and/or component of tested organism) and abiotic (ABA, salt, temperature changes, dark, light intensity, drought, oxidative stress) stimuli and also appearance of senescence symptoms. OE indicates overexpression, NA indicates-not available, WT- wild type.

GENES	ATG numbers	USED MUTANTS	BIOTIC STRESS	ABIOTIC STRESS	reference
BAK1	AT4G33430	*bak1-5; bak1-3, bak1-4*	sensitive to *A.brassicicola*	insensitive to ABA	[73,74,160]
			sensitive to *P.syringae*		
		OEBAK1	NA	NA	
SERK2	AT1G34210	*serk2*	sensitive to *S.sclerotiorum*	sensitive to salt	[79,80]
				sensitive to ABA	[80]
		OESERK2	NA	tolerant to salt	[80]
SERK4	AT2G13790	*serk4; serk4-1*	weakly sensitive to BR treatment	early senescence	[20,81,161]
			sensitive to flg22 treatment		
		OESERK4	NA	delay senescence	[81]
BRI1	AT4G39400	*bri1; bri1-5*	disease resistant	sensitive to cold	
				sensitive to ABA	[74]
				sensitive to dark, light	[162]
		OEBRI	NA	sensitive to ABA on seed germination stage	[162]
BIR1	AT5G48380	*bir1-1*	resistant to biotrophic oomycete	sensitive to temperature change	[72,163]
		OEBIR1	NA	NA	
EFR	AT5G20480	*efr*	sensitive to *Agrobacterium*	NA	[98]
		OEEFR	NA	NA	
SOBIR1	AT2G31880	*sobir1*	insensitive to nlp20 treatment	NA	[13]
			sensitive to *S.sclerotiorum* and *B.cinerea*		
		OESOBIR1	NA	NA	
PSKR1	AT2G02220	*pskr1; pskr1-3*	sensitive to *A.brassicicola*	early senescence	[104]
			resistant to *P.syringae*		[164]
			sensitive to fungal elicitor E-Fol		[165]
		OEPSKR1	NA	delay senescence	[104]
PEPR1	AT1G73080	*pepr1*	NA	sensitive to salt stress	[106]

				insensitive to *At*Pep1-induced stomatal closure	[107]
		OEPEPR1	NA	NA	
LRR1	AT5G16590	*lrr1*	NA	sensitive to drought	[111]
		OELRR1	resistant to *P.syringae*	NA	[110]
			resistant to *Hyaloperonospora*		[110]
KIN7	AT3G02880	*kin7*	NA	sensitive to drought	[111]
				insensitive to increased CO2 level	[112]
		OEKIN7	NA	NA	
RLK902	AT3G17840	*rlk902*	sensitive to *P.syringae*	NA	[116]
			resistant to downy mildew		[115]
		OERLK902	resistant to *P.syringae*	NA	[116]
NIK1	AT5G16000	*nik1*	sensitive to geminivirus	NA	[166]
		OENIK1	resistant to kanamycin	NA	[167]
NIK3	AT1G60800	*nik3*	sensitive to geminivirus	NA	[117]
			sensitive to CaLCuV infection		[117]
		OENIK3	NA	NA	
FEI2	AT2G35620	*fei2*	sensitive to *B.cinerea*	NA	[118]
		OEFEI2	NA	NA	
RPK2	AT3G02130	*rpk2*	sensitive to nematode infection	NA	[128]
		OERPK2	NA	NA	
ERL1	AT5G62230	*erl1*	NA	sensitive to salt	[120]
		OEERL1	NA	NA	
PXL1	AT1G08590	*pxl1*	NA	sensitive to cold and heat	[122]
		OEPXL1	NA	insensitive to heat	[122]
PXL2	AT4G28650	*pxl2*	NA	sensitive to ABA	[121]
		OEPXL2	NA	NA	
LRR XI-23/ RLK7	AT1G09970	*lrr/rlk7*	NA	tolerant to H2O2 treatment	[127]
		OELRK7	NA	intolerant to H2O2 treatment	[127]

RPK1	AT1G69270	*rpk1*	NA	insensitive to ABA	[168]
		OERPK1	NA	tolerant to drought, oxidative stress	[169]
BRL3	AT3G13380	*brl3*	NA	sensitive to high glucose	[124]
		OEBRL3	NA	tolerant to drought	[125]
SARK	AT4G30520	*sark-1*	NA	delay leaf senescence	[130]
		OESARK	NA	early senescence	[130]
SGN3	AT4G20140	*sgn3*	NA	hypersensitive to low potassium	[123]
		OESGN3	NA	NA	

Table S2. Orthologous of stress related LRR-RLKs genes in *A.thaliana, O.sativa, G.max, M.truncatula, Populus, V. vinifera, S.lycopersicum, B.napus, Z.mays*. Orthologous of LRR-RLKs genes tested for different stress stimuli were taken from ATTED database (https://atted.jp/). The respective sequence of shown locuses for different plant species are in ATTED database.

Genes	*Plants*								
	A.thaliana	*O.sativa*	*G.max*	*M.truncatula*	*Populus*	*V.vinifera*	*S.lycopersicum*	*B.napus*	*Z.mays*
RKL1	RLK902	LOC4332106<br>LOC4333893	LOC100781597<br>LOC100788892<br>LOC100801582<br>LOC100818955	LOC11408124<br>LOC11435862<br>LOC25497283	LOC7474836<br>LOC7487396	LOC100243943<br>LOC100267283	LOC101247929<br>LOC101254257<br>LOC101268851	LOC103833000<br>LOC103838993<br>LOC103869641	LOC542104<br>LOC100285260<br>LOC100285980
PEPR2	PEPR1	LOC4345707<br>LOC4345708	LOC100778035<br>LOC100781454<br>LOC100802056<br>LOC100817655	LOC11415181<br>LOC25488017	LOC7486765<br>LOC7497791	LOC100258774	PORK1	LOC103830768<br>LOC103872555<br>LOC103872556	LOC100381406<br>LOC103643254
IRK	AT5G01890	LOC4327614<br>LOC4332757<br>LOC4339111	LOC100780307<br>LOC100791204<br>LOC100791629<br>LOC100813535	LOC11433630<br>LOC25489825	LOC7459241<br>LOC7482595<br>LOC7497359	LOC100248331<br>LOC100257029	LOC101247434<br>LOC101254001<br>LOC101262241	LOC103841545<br>LOC103850399	LOC100274594<br>LOC100304322<br>LOC103630489
FEI2	FEI1	LOC4332359	LOC100816028<br>LOC100819230	LOC11410978		LOC100264793	LOC101257290	LOC103857572<br>LOC103865464<br>LOC103867340	LOC100280440<br>LOC100281527
LRR XI-23/ RLK7	IKU2	LOC4352872	LOC100779554<br>LOC100809356<br>LOC100811489	LOC11418845<br>LOC25490156	LOC7469669	LOC100248069<br>LOC109121676	LOC101243702<br>LOC101255038	LOC103837012<br>LOC103843324<br>LOC103868127<br>LOC103871776	LOC103650295
PXY		LOC4344655	LOC100809695<br>LOC100813189	LOC25491892	LOC7465499	LOC100266344	LOC101256593<br>LOC101263386	LOC103860599	LOC100383417
PXL1	AT4G28650	LOC4328049<br>LOC4331599	LOC100779939<br>LOC100788430<br>LOC100789665<br>LOC100793873<br>LOC100811284<br>LOC100817339	LOC11411044<br>LOC11421682<br>LOC25484201	LOC7461486<br>LOC7487195<br>LOC7487717	LOC109122934	LOC101246467<br>LOC101249348	LOC103836383<br>LOC103854284	LOC103626517<br>LOC103631713<br>LOC103639559
PXL2	AT1G08590	LOC4328049<br>LOC4331599	LOC100779939<br>LOC100788430<br>LOC100789665<br>LOC100793873<br>LOC100811284<br>LOC100817339	LOC11411044<br>LOC11421682<br>LOC25484201	LOC7461486<br>LOC7487195<br>LOC7487717	LOC109122934	LOC101246467<br>LOC101249348	LOC103836383<br>LOC103854284	LOC103626517<br>LOC103631713<br>LOC103639559

BAM1	BAM2<br>BAM3	LOC4332141<br>LOC4334273<br>LOC4342345	RLK1<br>RLK3<br>RLK2<br>LOC100499646<br>LOC100777902<br>LOC100803075<br>LOC100815103<br>LOC100816158	LOC11408427<br>LOC11424811<br>LOC11431479<br>LOC25490539	LOC7465379<br>LOC7465545	LOC100258232<br>LOC100855393	LOC101248733<br>LOC101248913<br>LOC101264597	LOC103834155<br>LOC103858059<br>LOC103860625<br>LOC103861192<br>LOC103874010	LOC103644083<br>LOC100279272<br>LOC100383797<br>LOC109939609
SERK1	SERK4<br>SERK5<br>BAK1<br>SERK2	LOC4336035<br>LOC4344785	LOC100305355<br>SERK1<br>LOC100789884<br>LOC100800522<br>LOC100813918	LOC11421599<br>LOC11428529<br>LOC11430748<br>LOC25485006	LOC7478140	LOC100247340<br>LOC100247683<br>LOC100253855<br>LOC100266543	SERK3A<br>SERK1<br>SERK3B	LOC103830829<br>LRRII6<br>LOC103834483<br>LRRII5<br>LRRII4<br>LOC103862237	LOC542015<br>LOC542016<br>LOC542670<br>LOC103641205
CLV1		LOC4342080	NARK<br>CLV1A<br>LOC102664073	LOC11438702<br>LOC11439632	LOC7490636	LOC100260741	CLV1	LOC103832028	LOC103626458
CRN		LOC4326956	LOC102664172<br>LOC102664273	LOC11437782	LOC7491429	LOC104880871	LOC101055499	LOC103846585<br>LOC103850856<br>LOC103856008	LOC100285492
NIK1	NIK2	LOC4330602<br>LOC4340743	LOC100305388<br>LOC100790194<br>LOC100797768<br>LOC100798412<br>LOC100804283<br>LOC100806650	LOC25479984<br>LOC25495505<br>LOC25497639	LOC7466941<br>LOC7468388<br>LOC7483901	LOC100249555<br>LOC100253129	LOC101245114<br>LOC101246914<br>LOC101250403	LRRII2<br>LOC103856175<br>LOC103875371	LOC100382590<br>LOC100280233<br>LOC100281584
NIK3		LOC4327640<br>LOC9268978	LOC100784164<br>LOC100806943	LOC11407415	LOC7460771<br>LOC7469057	LOC100256748	LOC101245842<br>LOC101258453	LRRII1	LOC100272802<br>LOC100383570<br>LOC103635459
RLK902	RKL1	LOC4332106<br>LOC4333893	LOC100781597<br>LOC100788892<br>LOC100801582<br>LOC100818955	LOC11408124<br>LOC11435862<br>LOC25497283	LOC7474836<br>LOC7487396	LOC100243943<br>LOC100267283	LOC101247929<br>LOC101254257<br>LOC101268851	LOC103833000<br>LOC103838993<br>LOC103869641	LOC542104<br>LOC100285260<br>LOC100285980
EFR	AT3G47090<br>AT3G47110<br>AT3G47570<br>AT3G47580<br>AT5G39390	LOC4324494<br>LOC9266358<br>LOC9267517<br>LOC9269230 (<br>LOC9270033<br>LOC9271075	LOC100776429<br>LOC100777490<br>LOC100778093<br>LOC100782685<br>LOC100783512<br>LOC100785561	LOC11406338<br>LOC11406710<br>LOC11406756<br>LOC11407069<br>LOC11407070<br>LOC11407873	LOC7463459<br>LOC7463460<br>LOC7468441<br>LOC7469474<br>LOC7474826<br>LOC7476326	LOC100241667<br>LOC100258860<br>LOC100259050<br>LOC100259715<br>LOC100263845	LOC101246542<br>LOC101250857<br>LOC101251570<br>LOC101256934<br>LOC101257348<br>LOC101258336	LOC103832809<br>LOC103838692<br>LOC103839158<br>LOC103841098<br>LOC103841563<br>LOC103844376	LOC103626075<br>LOC103628995<br>LOC103634832<br>LOC103634833<br>LOC103640781<br>LOC103641369

	LOC9271462 (	LOC100786600	LOC11408622	LOC7482217	LOC100265904	LOC101261427	LOC103845674	LOC103649762
	LOC9271745	LOC100792724	LOC11408648	LOC7486319	LOC109122564	LOC101261723	LOC103848469	LOC103650269
	LOC9272400	LOC100801459	LOC11409452	LOC7494927		LOC101266316	LOC103848471	LOC103652739
	LOC4324497	LOC100813523	LOC11409458			LOC112940090	LOC103856446	LOC103652958
	LOC4324499	LOC102664380	LOC11410960				LOC103856633	LOC103653760
	LOC4324501	LOC102664710	LOC11411980				LOC103857204	LOC103653761
	LOC4328700	LOC106794146	LOC11413493				LOC103864894	LOC103653766
	LOC4328704		LOC11415807				LOC103864895	LOC103653770
	LOC4328722		LOC11415808				LOC103867613	LOC103653773
	LOC4328724		LOC11416344				LOC103868348	LOC103654972
	LOC4328725		LOC11416554				LOC103869459	LOC100272957
	LOC4329980		LOC11416555				LOC103873225	LOC109940253
	LOC4329982		LOC11418993				LOC103873260	LOC109946042
	LOC4329983		LOC11419156				LOC103873370	
	LOC4330078		LOC11421706					
	LOC4341380		LOC11423999					
	LOC4342285 (		LOC11426285					
	LOC4345065 (		LOC11426548					
	LOC4348346		LOC11428017					
	LOC4349906		LOC11430041					
	LOC4350541		LOC11432152					
	LOC4350749		LOC11438431					
	LOC4350958		LOC25482466					
	LOC107275420		LOC25482467					
	LOC107275524		LOC25484776					
	LOC107276008		LOC25484777					
	LOC107276079		LOC25484778					
	LOC107276445		LOC25486456					
	LOC107276510		LOC25487142					
	LOC107276755		LOC25487144					
	LOC107277108		LOC25487145					
	LOC112937978		LOC25496117					
			LOC25501419					
			LOC112422017					
			LOC112422018					
			LOC112422019					
			LOC112422020					

RPK2	RPK1	LOC4334156<br>LOC4343832	LOC100802574<br>LOC100803058<br>LOC100803891<br>LOC100819882	LOC11417156<br>LOC25495342	LOC7471063<br>LOC7472595<br>LOC7484575	LOC100254985<br>LOC100854554	LOC101259816	LOC103842915<br>LOC103852567<br>LOC103860116	LOC103625875<br>LOC103631887<br>LOC103633402
HAK1	AT5G37450	LOC4325243<br>LOC9267172	LOC100777308<br>LOC100786467<br>LOC100799888<br>LOC100801035	LOC11410297<br>LOC11413754<br>LOC11435322	LOC7465186<br>LOC7465190<br>LOC7482611<br>LOC7498114	LOC100245012<br>LOC100265706	LOC101252112<br>LOC101264274	LOC103844004<br>LOC103855391	LOC103630498<br>LOC103630507<br>LOC103632695<br>LOC103646897
SOBIR1		LOC4340783	LOC100788151<br>LOC100811980	LOC25489526	LOC7453813	LOC100248492	LOC101252072<br>LOC101259548	LOC103857405<br>LOC103865167<br>LOC103865168	LOC100274364
PSKR1	PSKR2	LOC4330050<br>LOC4337364<br>LOC4337367<br>LOC4342205	LOC100789534<br>LOC100791334<br>LOC100807582	LOC25487312		LOC100254716<br>LOC100260504	LOC101246169<br>LOC101258171	LOC103844858<br>LOC103852007<br>LOC103854012	LOC109942164<br>LOC103627437<br>LOC100272954
BIR1		LOC4338797<br>LOC4350208	LOC100305365<br>LOC100801294<br>LOC100817267	LOC11428918<br>LOC25500213	LOC7464590<br>LOC7495475	LOC100854149<br>LOC100251471	LOC101261424	LOC103828382<br>LOC103874952	LOC100281404<br>LOC100285625
SARK	AT2G23950	LOC4345699	LOC100775565<br>LOC100797186	LOC11425481	LOC7485269<br>LOC7493367	LOC100244999	LOC101254706	LOC103861972<br>LOC103864508	LOC103643259
BRI1		LOC4324691	BRI1B<br>BRI1A	LOC11441490	LOC7456279<br>LOC7477141	LOC100252647	CURL3	LOC103828047<br>LOC103835122<br>LOC103845289	LOC103636232<br>LOC103642983
BRL3	BRL1	LOC4346640<br>LOC9272003	BRL1B<br>BRL1A	LOC25488505		LOC100249741	LOC101258083	LOC103832569<br>LOC103870174	LOC103632518<br>LOC103653350
ER	ERL2<br>ERL1	LOC4330905<br>LOC4340000<br>LOC4340427	LOC100305428<br>LOC100305433<br>LOC100499643<br>LOC100782307<br>LOC100795363<br>LOC100800185<br>LOC100804875<br>LOC100811653	LOC11428771<br>LOC25485110	LOC7476769<br>LOC7482132	LOC100256609<br>LOC100259283	LOC101244640<br>LOC101266437	LOC103842153<br>LOC103847083<br>LOC103874661	LOC100383941<br>LOC100279811<br>LOC100193598
CEPR2		LOC4325091<br>LOC4350138	LOC100789704	LOC11435226	LOC7455276	LOC100260256	LOC101250763<br>LOC101253498	LOC103650929<br>LOC103652818	LOC103650929<br>LOC103652818
KIN7	AT5G16590							LOC103843628<br>LOC103846319<br>LOC103851054<br>LOC103870882	

LRR1	AT3G02880	LOC103843628<br>LOC103846319<br>LOC103851054<br>LOC103870882

Table S3. Genetic tools in different crop plants in investigation of stress-related LRR-RLKs. Generated mutants defective in LRR-RLK genes in *N. benthamiana, S.lycopersicum, S.tuberosum,* S.*commersonii, O.sativa, T.aestivum, H.vulgare, B.distachyon* were taken from different research studies about role of LRR-RLK genes. OE-overexpression; Ri or RNAi – silenced mutant line, TRV- tobacco rattle virus, VIGS- Virus-induced gene silencing, p-promoter.

LRR-RLK gene	***N. benthamiana***	***S.lycopersicum/ S. tuberosum/ S. commersonii***	***O. sativa***	***T. aestivum /H.vulgare /B. distachyon***	**Reference**
BRI	*TRV:NbBRI1*	*SlBRI1* OE; *cu3* mutant; *StBRI1* in *bri1-5*; *StBRI1* Ri mutant	OE anti-*OsBRI1*; *d61-4* mutant	*OETaBRI1* in *Arabidopsis*; site direct modification of *TaBRI1* (Cas9/gRNA); silencing (VIGS) of *BRI1*; *BdBRI1-RNAi* mutants	[83,86-89,91-94,126, 181]
EFR	*AtEFR* into tobacco	*AtEFR* into tomato ; *AtEFR* into potato	*EFR:XA21* chimera in *Arabidopsis*	*pActEFR* into wheat	[98,99,100,103, 170]
ER	-	*pER::ER* (At) genomic in tomato	*pER::ER* (At) genomic in rice	*vasc1–1* mutants	[171-174]
			Loss-of-function mutants of *OsER1, OsER2*		
CLV1	-	*SIclv1* mutant	-	-	[175,176]
*SOBIR*1	OE of *NbSOBIR1* in tobacco	OE of *SlSOBIR1* in tobacco	-	-	[13,18,95,96]
	TRV:NbSOBIR1	*TRV:SISOBIR1*			
	sobir1/sobir1-like				
BAK1	*NbSerk3A*	*SlSERK3A* silencing (VIGS)	Ectopic *AtBAK1*	-	[74-76,177]
	NbSerk3B	*SlSERK3B* silencing (VIGS)			
LRR1	-	-	*LRR1Ri*	*TaLRRK-6D/HvLRRK-6H*	[113,114,127]
SERK2	-	-	*OsSerk2Ri*	-	[77,79]
SERK1	-	-	*OEOsSerk1*	-	[78]
RLK1	-	-	*Ir-lrr (Ri)*	-	[178,179]
PEPR1	-	*PERK1* RNAi	-	-	[108, 180]
PSKR1	-	-	*OE OsPSKR1*	-	[104,105]
BRL3	-	-	D61-4 allele	-	[124,126]
ERL1	-	-	mutations in *OsERL*	-	[119,120]

Table S4 Potential interactions of stress-related LRR- RLKs (from LRR II family) with other LRR- RLKs. Formation of heterodimers were taken from ATTED database. The formation of heterodimers between the LRR- RLKs were labeled as "heterodimer". The inside box cross between two LRR- RLK genes; one from column and second from row. The method/s of identifying heterodimer formation was solid-phase assay. NA indicates data not available

	**Co-receptors (LRR II)**							
**LRR- RLK**	**SERK2**	**SERK1**	**SERK4/ BKK1**	**SERK3/ BAK1**	**SARK/Cik3**	**NIK1**	**NIK3/Cik1**	**Reference**
**IOS1**	*NA*	heterodimer (solid-phase assay)	heterodimer (solid-phase assay)	heterodimer (solid-phase assay)	heterodimer (solid-phase assay)	heterodimer (solid-phase assay)	*NA*	[152,153]
**PSY1R**	*NA*	heterodimer (solid-phase assay)	heterodimer (solid-phase assay)	heterodimer (solid-phase assay)	heterodimer (solid-phase assay)	heterodimer (solid-phase assay)	*NA*	[152,153]
**SRF6**	*NA*	heterodimer (solid-phase assay)	heterodimer (solid-phase assay)	heterodimer (solid-phase assay)	heterodimer (solid-phase assay)	heterodimer (solid-phase assay)	*NA*	[152,153]
**SRF7**	*NA*	heterodimer (solid-phase assay)	heterodimer (solid-phase assay)	heterodimer (solid-phase assay)	heterodimer (solid-phase assay)	heterodimer (solid-phase assay)	*NA*	[152,153]
**SRF8**	*NA*	heterodimer (solid-phase assay)	heterodimer (solid-phase assay)	heterodimer (solid-phase assay)	heterodimer (solid-phase assay)	heterodimer (solid-phase assay)	*NA*	[152,153]
**GHR1**	*NA*	heterodimer (solid-phase assay)	heterodimer (solid-phase assay)	heterodimer (solid-phase assay)	heterodimer (solid-phase assay)	heterodimer (solid-phase assay)	*NA*	[153]
**SRF2**	*NA*	*NA*	heterodimer (solid-phase assay)	heterodimer (solid-phase assay)	heterodimer (solid-phase assay)	heterodimer (solid-phase assay)	*NA*	[152,153]
**SRF3**	*NA*	*NA*	heterodimer (solid-phase assay)	heterodimer (solid-phase assay)	heterodimer (solid-phase assay)	heterodimer (solid-phase assay)	*NA*	[152,153]
**GSO1**	*NA*	*NA*	*NA*	heterodimer (solid-phase assay)	heterodimer (solid-phase assay)	heterodimer (solid-phase assay)	heterodimer (solid-phase assay)	[152,153]
**BRL3**	*NA*	*NA*	heterodimer (solid-phase assay)	heterodimer (solid-phase assay)	heterodimer (solid-phase assay)	heterodimer (solid-phase assay)	*NA*	[152,153]
**SARK/CIK3**	*NA*	heterodimer (solid-phase assay)	heterodimer (solid-phase assay)	heterodimer (solid-phase assay)	*NA*	heterodimer (solid-phase assay)	*NA*	[152]

**BRL1**	*NA*	*NA*	*NA*	heterodimer (anti tag CoIP, yeast two-hybrid assay)	heterodimer (solid-phase assay)	heterodimer (solid-phase assay)	*NA*	[17, 152]
**SERK5**	*NA*	*NA*	heterodimer (solid-phase assay)	heterodimer (solid-phase assay)	heterodimer (solid-phase assay)	*NA*	*NA*	[152,153]
**FEI1**	heterodimer (solid-phase assay)	*NA*	*NA*	heterodimer (solid-phase assay)	heterodimer (solid-phase assay)	*NA*	*NA*	[153]
**SRF5**	*NA*	*NA*	*NA*	heterodimer (solid-phase assay)	heterodimer (solid-phase assay)	heterodimer (solid-phase assay)	*NA*	[152,153]
**SRF1**	*NA*	*NA*	*NA*	heterodimer (solid-phase assay)	heterodimer (solid-phase assay)	heterodimer (solid-phase assay)	*NA*	[152,153]
**RLK902**	*NA*	*NA*	*NA*	heterodimer (solid-phase assay)	heterodimer (solid-phase assay)	NA	heterodimer (solid-phase assay)	[152,153]
**NIK2**	*NA*	*NA*	*NA*	heterodimer (solid-phase assay)	heterodimer (solid-phase assay)	NA	NA	[152,153]
**PRK1**	*NA*	*NA*	*NA*	heterodimer (solid-phase assay)	*NA*	heterodimer (solid-phase assay)	NA	[153]
**NIK3**	heterodimer (solid-phase assay)	*NA*	*NA*	heterodimer (solid-phase assay)	NA	NA	NA	[152,153]
**PRK6**	*NA*	*NA*	*NA*	heterodimer (solid-phase assay)	*NA*	*NA*	heterodimer (solid-phase assay)	[152,153]
**PRK2A**	*NA*	*NA*	*NA*	*NA*	*NA*	*NA*	heterodimer (solid-phase assay)	[153]
**PRK4**	*NA*	*NA*	*NA*	*NA*	*NA*	*NA*	heterodimer (solid-phase assay)	[152]
**PRK5**	*NA*	*NA*	*NA*	*NA*	*NA*	*NA*	heterodimer (solid-phase assay)	[153]
**MRLK**	*NA*	*NA*	*NA*	*NA*	*NA*	*NA*	heterodimer (solid-phase assay)	[153]
**BRL2**	heterodimer (solid-phase assay)	*NA*	*NA*	*NA*	*NA*	*NA*	*NA*	[153]
